# Chemical constituents from the aerial parts of *Euphorbia sikkimensis* and their bioactivities

**DOI:** 10.1007/s13659-013-0006-y

**Published:** 2013-05-23

**Authors:** Da-Song Yang, Wei-Bing Peng, Zi-Lei Li, Xue Wang, Jian-Guo Wei, Ke-Chun Liu, Yong-Ping Yang, Xiao-Li Li

**Affiliations:** 16 Key Laboratory of Economic Plants and Biotechnology, Kunming Institute of Botany, Chinese Academy of Sciences, Kunming, 650201 China; 26 Plant Germplasm and Genomics Center, the Germplasm Bank of Wild Species, Kunming Institute of Botany, Chinese Academy of Sciences, Kunming, 650201 China; 36 Institute of Tibetan Plateau Research at Kunming, Kunming Institute of Botany, Chinese Academy of Sciences, Kunming, 650201 China; 46 Biology Institute of Shandong Academy of Sciences, Jinan, 250014 China

**Keywords:** *Euphorbia sikkimensis*, ingenol, trinortriterpenoid, tocopherol derivatives, bioactivities

## Abstract

Phytochemical investigation of the aerial parts of *Euphorbia sikkimensis* led to the isolation of one new diterpenoids, named sikkimenoid E (**1**), together with thirteen other known compounds (**2–14**). Their structures were established by means of spectroscopic methods. Compound **2** was identified to be a trinortriterpenoid, and derived for the first time from a natural source. In this paper we reveal for the first time its comprehensive spectral data and NMR spectral assignment. Compound **4** showed antiangiogenic activity with an IC_50_ value of 5.66 µM in a zebrafish model, and compounds **5** and **6** exhibited cytotoxicity toward A549 cell line with IC_50_ values of 12.12 and 6.45 µM, respectively.

**Electronic Supplementary Material:**

Supplementary material is available for this article at 10.1007/s13659-013-0006-y and is accessible for authorized users.

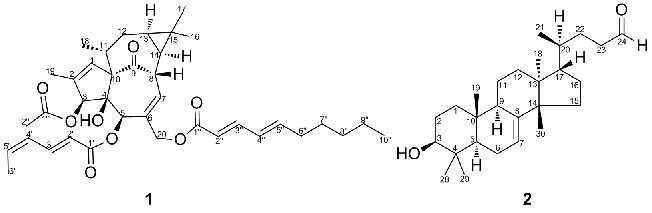

## Introduction

Plants of the genus *Euphorbia* are well known for their chemical diversity of their isoprenoid constituents. Terpenoids with different core frameworks perform extensive activities, such as anti-proliferation, modulability of multidrug resistance, cytotoxic activity, antimicrobial and anti-inflammatory bioactivities.[1] Also, the roots of *Euphorbia sikkimensis* Boiss have been used in traditional Chinese medicine, for the treatment of poisoning, malaria, rheumatism, and other disorders.[2] Previous studies on this plant have resulted in the isolation of four jatropholane-type diterpenoids.[3] Our continuing phytochemical investigation on the aerial parts of *E. sikkimensis* led to the isolation of a new diterpenoid, named sikkimenoid E (**1**), along with thirteen known compounds (**2**–**14**). Compound **2** was revealed as a trinortriterpenoid derived from the oxidation of 3*S*,24*S*,25-trihydroxytirucall-7-ene, and until recently, only its mass spectrometry data had been reported.[4] Other known compounds were identified as 8-geranyloxypsolaren (**3**),[5] (–)-bornyl ferulate (**4**),[6] isopimara-8(14),15-dien-3-one (**5**),[7] 10-hydroxydepressin (**6**),[8]

**Figure 1.** The structures of compounds **1**–**14**

*To whom correspondence should be addressed. E-mail: li_xiaoli11@mail.kib.ac.cn cycloart-23*E*-ene-3*β*,25-diol (**7**),[9] sericeol (**8**),[10] spiroinonotsuoxodiol (**9**),[11] *α*-tocopherolquinone (**10**),[12] (2*R*,4a*R*,8a*R*)-3,4,4a,8a-tetrahydro-4a-hydroxy-2,6,7,8a-tetramethyl-2-(4,8,12-trimethyltridecyl)-2*H*-chromene-5,8-dione (**11**),[13] *α*-tocopherol (**12**),[14] 5-methoxymethyl-7,8-dimethyltocol (**13**),[15] and peplusol (**14**).[16] All isolated compounds were evaluated for their anti-angiogenic activities using a zebrafish model and also for their cytotoxic potential against human lung cancer cells A549. In this paper, we report the isolation, structure elucidation and biological activities of these compounds.

## Results and Discussion

Compound **1** was obtained as an optically active colorless oil ($[\alpha]_D^{22}$ + 37.5) and the molecular formula was deduced to be $C_{38}H_{50}O_8$ based on its HREIMS data (*m/z* 634.3518, calcd 634.3506, $[M]^+$), suggesting 14 degrees of unsaturation. The UV spectrum displayed maximum absorption at 266 nm, which indicated the presence of conjugated chromophores. The IR spectrum of **1** suggested characteristic bands of hydroxyl (3442 $cm^{-1}$), carbonyl (1728 $cm^{-1}$) and olefinic (1640 $cm^{-1}$) groups. Analysis of the NMR spectra of **1** (Table 1) suggested the presence of one ketone ($\delta_C$ 205.7), four oxygen-bearing carbons ($\delta_C$ 86.0, 82.4, 77.0, and 66.1), three ester carbonyls ($\delta_C$ 172.6, 170.6, and 170.5), six pairs of double bonds, six methylenes (one oxygenated at $\delta_C$ 66.1) and seven methyls. Except for one ketone, six pairs of double bonds and three ester carbonyls, there should have been four rings in **1** to fit the 14 degrees of unsaturation. Comparison of the NMR spectra (Table 1) of **1** with those of 20-*O*-(2'*E*,4'*E*-decadienoyl)-ingenol[17] revealed that **1** has the typical signals of an ingenol skeleton, a common chemotype in genus *Euphobia*[1]. Further analysis of the 2D NMR of **1** (Figure 2) showed the $^1$H-$^1$H COSY correlations of H-7/H-8/H-14/H-13/H-12/H-11/Me-18 and HMBC correlations of H-1 with C-3, C-4, C-9 and Me-19; and of H-3 with C-2 and C-10; and of H-7 with C-5, C-6 and C-9; of H-12 with C-10 and C-15 also supported the existence of ingenol skeleton in **1**. The differences between **1** and 20-*O*-(2'*E*,4'*E*-decadienoyl)-ingenol could be rationalized to the carbon signals corresponding to acid moiety. The EIMS fragment peaks at *m/z* 151 $[C_9H_{15}CO]^+$, 95 $[C_5H_7CO]^+$, 354 $[M - C_9H_{15}COOH - C_5H_7COOH]^+$, 294 $[M - C_9H_{15}COOH - C_5H_7COOH - CH_3COOH]^+$ suggested the ester residues of compound **1** were 2,4-decadienoyloxy group, 2,4-hexadienoyloxy group and acetoxy group. The HMBC (Figure 2) correlations of H-3 with C-1''', and of H-20 with C-1'' demonstrated that the acetoxy and decadienoyloxy groups were located at C-3 and C-20, respectively. The proton signal of H-5 in **1** resonated at $\delta$ 5.45 (s), shifting downfield by 1.74 ppm, suggested that the hexadienoyloxy group is located at C-5. The configurations of conjugated double bonds were elucidated by analysis of its ROESY spectrum, comparison of the chemical shifts, and the coupling patterns with those reported data. In compounds with *Z,E*-configuration the coupling constants are normally $J_{2,3}$ = 11.3 Hz,[18] while coupling constants of *E,E* or *E,Z*-configuration are normally $J_{2,3}$ = 15.2 Hz.[17] In the case of **1**, $J_{2',3'}$ = 15.3 Hz, $J_{2'',3''}$ = 15.2 Hz, corresponding to a *trans* double bond between C-2' and C-3', and between C-2'' and C-3''. In addition, another difference between *E,E* and *E,Z*-configuration of conjugated double bonds was the chemical shifts of H-5, normally $\delta_{H-5}$ of *E,Z*-configuration was about 5.90 ppm, while $\delta_{H-5}$ of *E,E*-configuration was about 6.20 ppm.[17,18] Chemical shifts of 5' ($\delta_H$ 5.89) and 5'' ($\delta_H$ 6.19) of **1** indicating that double bond between C-4' and C-5' was *cis*, and that of between C-4'' and C-5'' was *trans*, which was also support by the observed ROESY correlation of H-4' with H-5' (Figure 3). The observed ROESY correlations of H-8 with Me-17; of H-13 with H-14; of Me-16 with H-13 and H-14; of H-13 with Me-18 and of Me-18 with H-1 suggested that the stereochemistry of ingenane diterpenoid part in **1** was the same as ingenol-3,5,20-triacetate, which was established by the single-crystal X-ray crystallography.[19] Therefore, the structure of **1** was determined as 3-*O*-acetyl-5-*O*-(2'*E*,4'*Z*-hexadienoyloyl)-20-*O*-(2''*E*,4''*E*-

**Figure 2.** Selected HMBC ( ) and $^1$H-$^1$H COSY ( ) correlations of compounds **1** and **2**

**Table 1. The NMR [150 ($^{13}$C) and 600 ($^1$H) MHz, CDCl$_3$, *δ* in ppm, *J* in Hz] data of 1**

| position | $\delta_C$ | $\delta_H$ | position | $\delta_C$ | $\delta_H$ |
|---|---|---|---|---|---|
| 1 | 132.2 CH | 6.07 (s) | 20a | 66.1 CH$_2$ | 4.43 (d, 12.5) |
| 2 | 133.4 C | | 20b | | 4.23 (d, 12.5) |
| 3 | 82.4 CH | 4.97 (s) | 1' | 170.5 C | |
| 4 | 86.0 C | | 2' | 117.3 CH | 5.88 (d, 15.3) |
| 5 | 77.0 CH | 5.45 (s) | 3' | 147.4 CH | 7.34 (dd, 15.3, 9.7) |
| 6 | 135.7 C | | 4' | 128.3 CH | 6.17 (m) |
| 7 | 131.3 CH | 6.20 (d, 4.2) | 5' | 143.1 CH | 5.89 (m) |
| 8 | 43.6 CH | 4.26 (m) | 6' | 20.9 CH$_3$ | 1.94 (br s) |
| 9 | 205.7 C | | 1'' | 170.6 C | |
| 10 | 71.9 C | | 2'' | 119.3 CH | 5.97 (d, 15.2) |
| 11 | 38.5 CH | 2.51 (m) | 3'' | 141.8 CH | 7.69 (dd, 15.2, 12.0) |
| 12*α* | 31.1 CH$_2$ | 2.27 (m) | 4'' | 126.3 CH | 6.13 (m) |
| 12*β* | | 1.74 (m) | 5'' | 146.4 CH | 6.19 (m) |
| 13 | 23.0 CH | 0.69 (dd, 15.6, 8.4) | 6'' | 33.0 CH$_2$ | 2.14 (q, 6.9) |
| 14 | 22.9 CH | 0.94 (m) | 7'' | 28.3 CH$_2$ | 1.40 (m) |
| 15 | 24.4 C | | 8'' | 31.4 CH$_2$ | 1.27 (m) |
| 16 | 28.4 CH$_3$ | 1.04 (s) | 9'' | 22.5 CH$_2$ | 1.28 (m) |
| 17 | 15.5 CH$_3$ | 1.08 (s) | 10'' | 14.0 CH$_3$ | 0.87 (t, 6.8) |
| 18 | 17.1 CH$_3$ | 0.98 (d, 7.1) | 1''' | 172.6 C | |
| 19 | 15.6 CH$_3$ | 1.75 (m) | 2''' | 21.2 CH$_3$ | 2.09 (s) |

decadienoyloyl)ingenol (Figure 1) and was given a trivial name sikkimenoid E.

Compound **2**, was obtained as a white powder, and showed a negative specific rotation ($[\alpha]_D^{25}$ – 16.2). The IR spectrum showed absorption bands at 3434 and 1639 $cm^{-1}$, revealing the existence of hydroxyl and olefinic groups. The $^1H$ and $^{13}C$ NMR data (Table 2) exhibited resonances for a trisubstituted double bond ($\delta_H$ 5.23, d, $J$ = 3.1 Hz; $\delta_C$ 118.0, d and 145.6, s), an aldehyde group ($\delta_C$ 203.1, d), an oxygenated methine ($\delta_H$ 3.22, dd, $J$ = 11.4, 4.0 Hz; $\delta_C$ 79.2, d). Comparison of the NMR data of **2** with those of cornusalterin J suggested that they share the same lanostane skeleton.[20] The differences could be rationalized to the changes of the side chain, of which the structural part from C-23 to C-27 in cornusalterin J was replaced by a methylene and an aldehyde group in **2**. This deduction was confirmed by the $^1H,^1H$-COSY correlation of H-22 with H-23 and HMBC correlations of both H-22 and H-23 with C-24, and of H-24 with C-22 (Figure 2). The observed ROESY correlations of H-3/H-5, H-5/Me-29, H-5/H-9, H-9/Me-18 and Me-18/H-20 indicated that H-3, H-5, H-9, Me-18, H-20 and Me-29 are cofacial and assigned to be $\alpha$-oriented, the same with the cornusalterin J. In turn the cross-peaks of Me-19/Me-28, Me-30/H-17, H-17/Me-21 indicated the $\beta$-oriention of H-17, Me-19, Me-21, Me-28 and Me-30. Thus, the structure of **2** was determined as shown. From a literature research, compound **2** was only recorded in one reference, which was derived from oxidation of 3*S*,24*S*,25-trihydroxytirucall-7-ene.[4] Therefore, this is first report of **2** found from a nature source and given a trivial name sikkimenoid F.

**1** **2**

**Figure 3.** Key ROESY correlations of compounds **1** and **2**

All the compounds were tested for their cytotoxicity against the human lung cancer cells A549 by the MTT method, with 5-FU used as a positive control ($IC_{50}$ 17.28 $\mu$M).[21] Compounds **5** and **6** exhibited cytotoxicity toward A549 cell line with $IC_{50}$ values of 12.12 and 6.45 $\mu$M, respectively. In addition, the anti-angiogenic activities of all compounds were further evaluated using a zebrafish model in terms of the inhibition of the growth of intersegmental vessels, using PTK787 as a positive control ($IC_{50}$ 0.23 $\mu$M).[22] The results showed that intersegmental vessels of embryo treated with compound **4** were significantly fewer than those of the control (0.2% DMSO in sterile salt water), and the reduction was dose dependent, and with an $IC_{50}$ value of 5.66 $\mu$M. It's the first time that the anti-angiogenic activity of compound **4** and the cytotoxicities of compounds **5** and **6** against A549 cell line were reported.

## Experimental Section

**General Experimental Procedures.** Optical rotations were measured on a JASCO P-1020 digital polarimeter. UV spectra were obtained using a Shimadzu UV-2401A spectrophotometer. IR spectra were obtained on a Bruker Tenor 27 spectrometer with KBr pellets. 1D and 2D NMR spectra were recorded on Bruker AM-400, DRX-500 or AV III-600 spectrometers with TMS used as an internal standard. ESIMS spectra were performed on a Finnigan MAT 90 instrument, EI and HREI spectra were recorded on a Waters AutoSpec Premier P776 instrument. Column chromatography was performed on Sephadex LH-20 (GE Healthcare), silica gel (200-300 mesh, Qingdao Marine Chemical Ltd., Qingdao, China), RP-18 gel (LiChroprep, 40–63 $\mu$m; Merck, Darmstadt, Germany), and MCI gel CHP 20P (75–150 $\mu$m, Mitsubishi Chemical Corporation, Tokyo, Japan). Semipreparative HPLC was performed on a Hewlett-Packard instrument (column: Zorbax SB-C18, 250 × 9.4 mm; DAD detector). Fractions were monitored by TLC, visualized by heating silica gel plates sprayed with 15% $H_2SO_4$ in EtOH.

**Plant Material.** The aerial parts of *E. sikkimensis* were collected from Gongbo Gyamda County of the Tibetan autonomous region of China in 2010, and identified by Professor Yong-Ping Yang. A voucher specimen (Yangyp-20100936) has been deposited at the Herbarium of Kunming Institute of Botany, Chinese Academy of Sciences.

**Extraction and Isolation.** The dried and powdered aerial parts of *E. sikkimensis* (11 kg) were extracted with 90% EtOH

**Table 2. The NMR [100 ($^{13}C$) and 400 ($^1H$) MHz, $CDCl_3$, $\delta$ in ppm, $J$ in Hz] data of 2**

| position | $\delta_C$ | $\delta_H$ | position | $\delta_C$ | $\delta_H$ |
|---|---|---|---|---|---|
| 1$\alpha$ | 37.1 $CH_2$ | 1.10 (td, 12.9, 3.8) | 14 | 51.2 C | |
| 1$\beta$ | | 1.65 (m) | 15$\alpha$ | 33.8 $CH_2$ | 1.47 (m) |
| 2$\alpha$ | 27.0 $CH_2$ | 1.56 (m) | 15$\beta$ | | 1.78 (m) |
| 2$\beta$ | | 1.65 (m) | 16$\alpha$ | 28.4 $CH_2$ | 1.24 (m) |
| 3 | 79.2 CH | 3.22 (dd, 11.4, 4.0) | 16$\beta$ | | 1.92 (m) |
| 4 | 38.9 C | | 17 | 53.0 CH | 1.48 (m) |
| 5 | 50.5 CH | 1.28 (dd, 12.0, 5.6) | 18 | 22.0 $CH_3$ | 0.80 (s) |
| 6$\alpha$ | 23.9 $CH_2$ | 1.92 (m) | 19 | 13.1 $CH_3$ | 0.72 (s) |
| 6$\beta$ | | 2.10 (m) | 20 | 35.5 CH | 1.44 (m) |
| 7 | 118.0 CH | 5.23 (d, 3.1) | 21 | 18.0 $CH_3$ | 0.82 (d, 6.3) |
| 8 | 145.6 C | | 22 | 27.6 $CH_2$ | 1.23 (m) |
| 9 | 48.8 CH | 2.15 (m) | 23 | 41.5 $CH_2$ | 2.40 (m) |
| 10 | 34.9 C | | 24 | 203.1 CH | 9.75 (br. s) |
| 11 | 18.4 $CH_2$ | 1.49 (m) | 28 | 27.6 $CH_3$ | 0.95 (s) |
| 12$\alpha$ | 33.7 $CH_2$ | 1.42 (m) | 29 | 14.7 $CH_3$ | 0.83 (s) |
| 12$\beta$ | | 1.63 (m) | 30 | 27.2 $CH_3$ | 0.94 (s) |
| 13 | 43.5 C | | | | |

(3 × 40 L) for 24 h at room temperature and filtrated. The filtrate was concentrated and partitioned between $H_2O$ and EtOAc and then the EtOAc portion was decolorized on MCI gel CHP 20P (eluting with 95% EtOH). The residue (690 g) was chromatographed on silica gel (80–100 mesh), eluting with $CHCl_3$-$Me_2CO$ (from 1:0 to 1:0.2), to derive fractions A–C. Fraction A was purified over a Sephadex LH-20, eluted with $CHCl_3$-MeOH (1:1) and then fractionated by RP-18 gel, eluted with MeOH-$H_2O$ (from 30% to 100%) to provide subfractions (A1–A6). These subfractions were repeatedly chromatographed on silica gel and Sephadex LH-20 respectively to yield compounds **3** (3.1 mg), **4** (22.3 mg), **5** (7.8 mg), **7** (5.3 mg), **11** (5.2 mg), **12** (20.0 mg), **13** (4.3 mg) and **14** (45.5 mg). Fraction C was chromatographed on RP-18 gel, eluted with a gradient of MeOH-$H_2O$ to afford five subfractions (C1–C5). C2 was further chromatographed on silica gel and Sephadex LH-20, and then purified by semipreparative HPLC (MeOH-$H_2O$, 70:30) to furnish **1** (2.6 mg, $t_R$ = 36 min) and **6** (5.2 mg, $t_R$ = 45 min). Compounds **2** (30.5 mg), **8** (12.7 mg), **9** (10.0 mg) and **10** (11.2 mg) were isolated from C3–C5 by repeatedly chromatographed on silica gel and Sephadex LH-20.

**Sikkimenoid E (1):** colorless oil; $[\alpha]_D^{22}$ + 37.5 (*c* 0.20, MeOH); UV (MeOH) $\lambda_{max}$ (log $\varepsilon$) 266 (4.18), 204 (4.33) nm; IR (KBr) $\nu_{max}$ 3442, 3425, 3398, 2957, 2928, 2871, 1728, 1640, 1461, 1380, 1314, 1234, 1156, 1131, 1024, 988 $cm^{-1}$; $^1H$ and $^{13}C$ NMR data see Table 1; EIMS *m/z* 634 $[M]^+$ (9), 522 (5), 372 (5), 354 (14), 312 (23), 294 (29), 233 (53), 151 (100), 122 (34), 95 (27), 81 (57); ESIMS *m/z* 657 $[M + Na]^+$; HREIMS *m/z* 634.3518 ($[M]^+$, calcd for $C_{38}H_{50}O_8$, 634.3506).

**Sikkimenoid F (2):** white powder; $[\alpha]_D^{25}$ − 16.2 (*c* 0.33, MeOH); UV (MeOH) $\lambda_{max}$ (log $\varepsilon$) 205 (3.64) nm; IR (KBr) $\nu_{max}$ 3434, 2951, 2931, 2881, 2716, 1724, 1639, 1464, 1384, 1276, 1248, 1163, 1100, 1066, 1034, 986 $cm^{-1}$; $^1H$ and $^{13}C$ NMR data see Table 2; EIMS *m/z* 400 ($[M]^+$).

**Cytotoxicity Assay.**[21] Compounds **1**–**14** were tested for their cytotoxicity against human lung cancer cell line A549 by the MTT method, and 5-FU was used as a positive control. Briefly, 100 $\mu$L cell suspension (1 × $10^5$ cells/mL) was seeded into 96-well microtiter plates and cultured for 24 h before the compound was added. Then, different concentrations of the compounds were added to the plates, the cells were cultivated for 48 h, and 10 $\mu$L of MTT (5 mg/mL) was added to each well. After 4 h, the culture medium was removed and the formazan crystal was completely dissolved with 150 $\mu$L DMSO to each well by vigorously shaking the plate. Finally, formazan absorbance was assessed by a BioRad microplate reader at 570 nm.

**Antiangiogenesis Assay.**[22] Stock solutions (20 mg/mL) of all samples were prepared by dissolving the test compounds in 100% DMSO. These solutions were diluted in sterile salt water (5 mM NaCl, 0.17 mM KCl, 0.4 mM $CaCl_2$, 0.16 mM $MgSO_4$) to obtain final solutions of various concentrations in 0.2% DMSO. Aliquots were placed into 24-well plates, and embryos (TG[VEGFR2:GRCFP]) at 24 hpf (hours postfertilization) were also transferred randomly into the above wells. Control embryos were treated with the equivalent amount of DMSO solutions. All embryos were incubated at 28.5 °C. After 48 h treatment, the intersegmental vessels of embryos were visualized with green fluorescent protein labeling and endogenous alkaline phosphatase staining. The antiangiogenic activities of compounds were calculated from the inhibition ratio of anti-angiogenesis.

## Electronic Supplementary Material

Supplementary material is available in the online version of this article at http://dx.doi.org/10.1007/s13659-013-0006-y and is accessible for authorized users.

## Acknowledgments

This work was supported by the Basic Research Project of the Ministry of Science and Technology of China (2012FY110300), MOST grant (2008BAD98B06).

**Open Access** This article is distributed under the terms of the Creative Commons Attribution License which permits any use, distribution, and reproduction in any medium, provided the original author(s) and source are credited.

## Electronic supplementary material

Supplementary material, approximately 1.82 MB.
