# Supplementary material for: Chemical constituents from the aerial parts of Euphorbia sikkimensis and their bioactivities
Source: Nat Prod Bioprospect. 2013 May 23;3(3):112–6. doi: 10.1007/s13659-013-0006-y (PMC4131670; doi:10.1007/s13659-013-0006-y)
Supplement: Supplementary file 1 — Supplementary material, approximately 1.82 MB. [file 13659_2013_6_MOESM1_ESM.pdf]

**Electronic Supplementary Material**

# Chemical constituents from the aerial parts of *Euphorbia sikkimensis* and their bioactivities

Da-Song YANG,[a,b,c] Wei-Bing PENG,[d] Zi-Lei LI,[a,b,c] Xue WANG,[d] Jian-Guo WEI,[a,b,c] Ke-Chun LIU,[d] Yong-Ping YANG,[a,b,c] Xiao-Li LI[a,b,c,]*

[a]Key Laboratory of Economic Plants and Biotechnology, Kunming Institute of Botany, Chinese Academy of Sciences, Kunming 650201, China

[b]Plant Germplasm and Genomics Center, the Germplasm Bank of Wild Species, Kunming Institute of Botany, Chinese Academy of Sciences, Kunming 650201, China

[c]Institute of Tibetan Plateau Research at Kunming, Kunming Institute of Botany, Chinese Academy of Sciences, Kunming 650201, China

[d]Biology Institute of Shandong Academy of Sciences, Jinan 250014, China

Received 22 January 2013; Accepted 28 March 2013

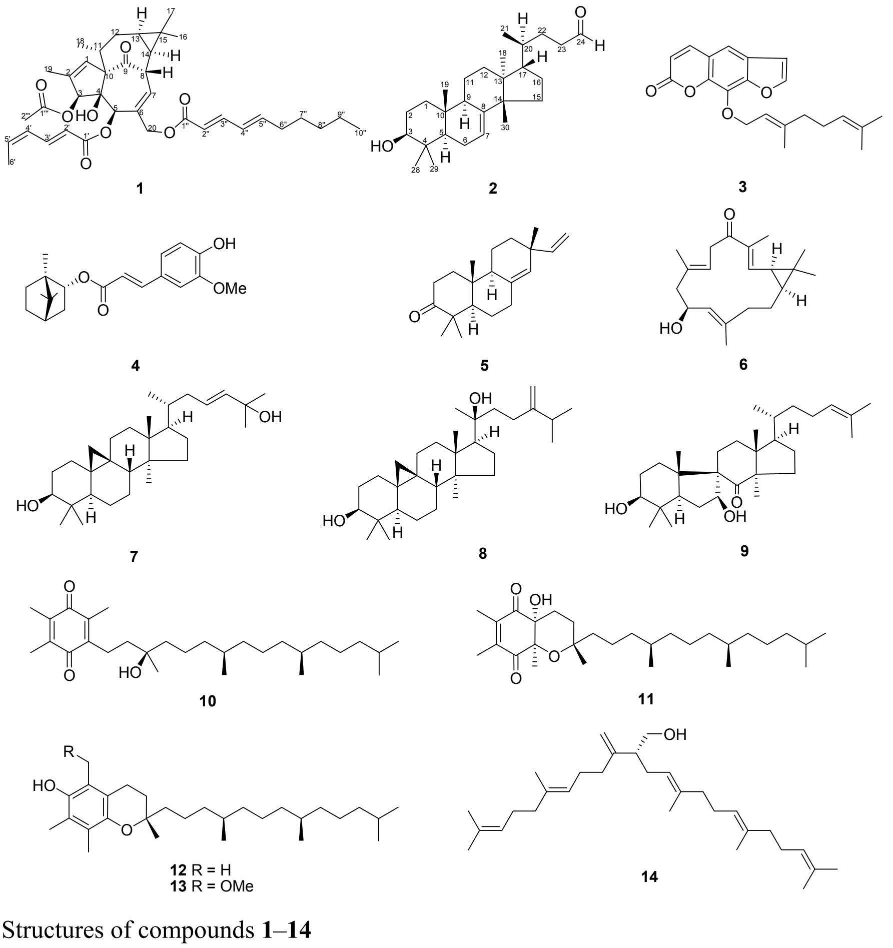

Structures of compounds **1**–**14**

*To whom correspondence should be addressed. E-mail: li_xiaoli11@mail.kib.ac.cn

# Contents of Electronic Supplementary Material


| No. | Contents | Page | No. | Contents | Page |
|---|---|---|---|---|---|
| 1 | Figure S1. $^{1}$H NMR spectrum of sikkimenoid E (**1**) | 3 | 12 | Figure S12. ORD spectrum of sikkimenoid E (**1**) | 14 |
| 2 | Figure S2. $^{13}$C NMR spectrum of sikkimenoid E (**1**) | 4 | 13 | Figure S13. $^{1}$H NMR spectrum of sikkimenoid F (**2**) | 15 |
| 3 | Figure S3. HSQC spectrum of sikkimenoid E (**1**) | 5 | 14 | Figure S14. $^{13}$C NMR spectrum of sikkimenoid F (**2**) | 16 |
| 4 | Figure S4. HMBC spectrum of sikkimenoid E (**1**) | 6 | 15 | Figure S15. HSQC spectrum of sikkimenoid F (**2**) | 17 |
| 5 | Figure S5. $^{1}$H–$^{1}$H COSY spectrum of sikkimenoid E (**1**) | 7 | 16 | Figure S16. HMBC spectrum of sikkimenoid F (**2**) | 18 |
| 6 | Figure S6. ROESY spectrum of sikkimenoid E (**1**) | 8 | 17 | Figure S17. $^{1}$H–$^{1}$H COSY spectrum of sikkimenoid F (**2**) | 19 |
| 7 | Figure S7. ESIMS spectrum of sikkimenoid E (**1**) | 9 | 18 | Figure S18. ROESY spectrum of sikkimenoid F (**2**) | 20 |
| 8 | Figure S8. EIMS spectrum of sikkimenoid E (**1**) | 10 | 19 | Figure S19. EIMS spectrum of sikkimenoid F (**2**) | 21 |
| 9 | Figure S9. HREIMS spectrum of sikkimenoid E (**1**) | 11 | 20 | Figure S21. IR spectrum of sikkimenoid F (**2**) | 22 |
| 10 | Figure S10. IR spectrum of sikkimenoid E (**1**) | 12 | 21 | Figure S22. UV spectrum of sikkimenoid F (**2**) | 23 |
| 11 | Figure S11. UV spectrum of sikkimenoid E (**1**) | 13 | 22 | Figure S24. ORD spectrum of sikkimenoid F (**2**) | 24 |

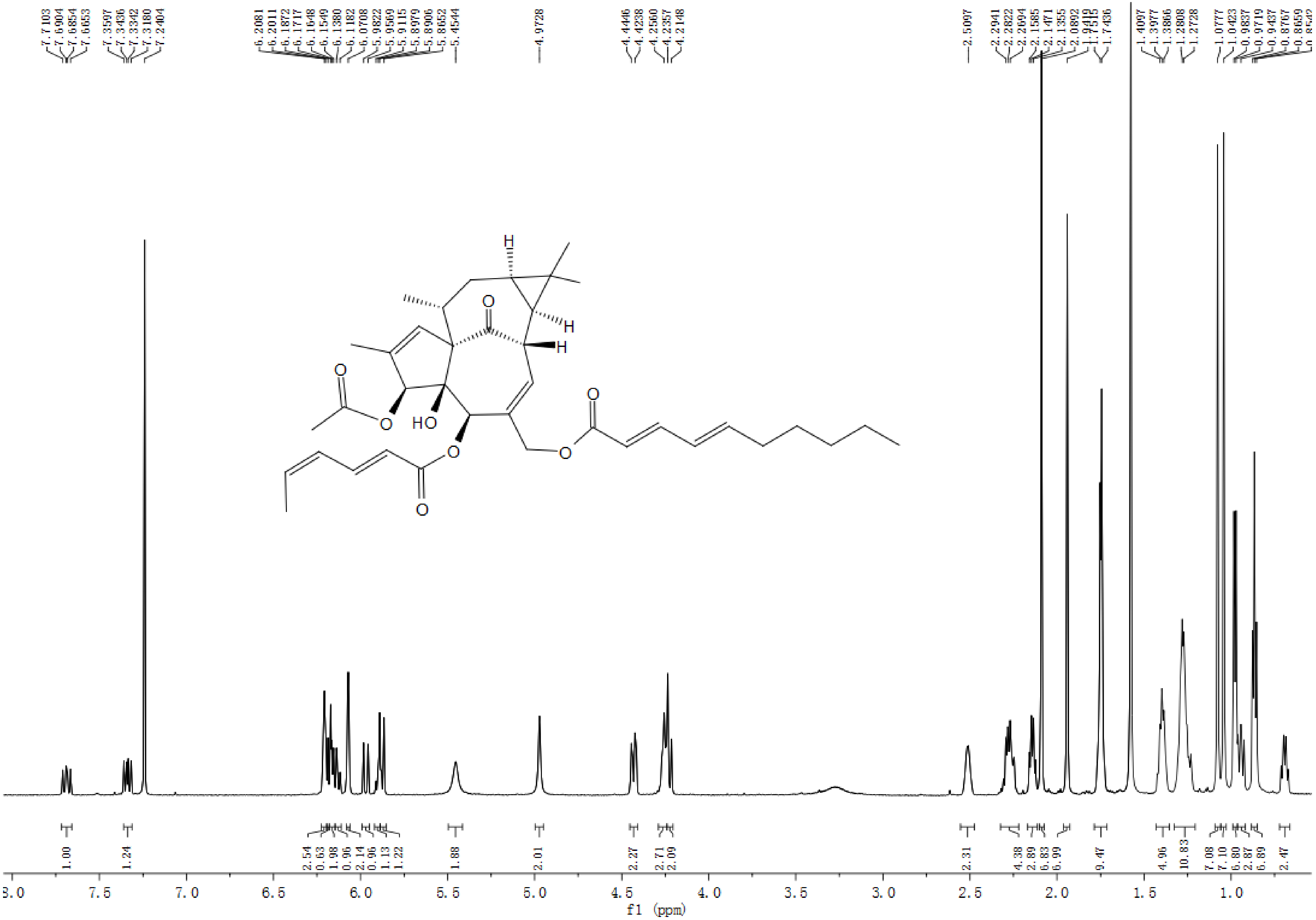


Figure S1. $^{1}$H NMR spectrum of sikkimenoid E (**1**)

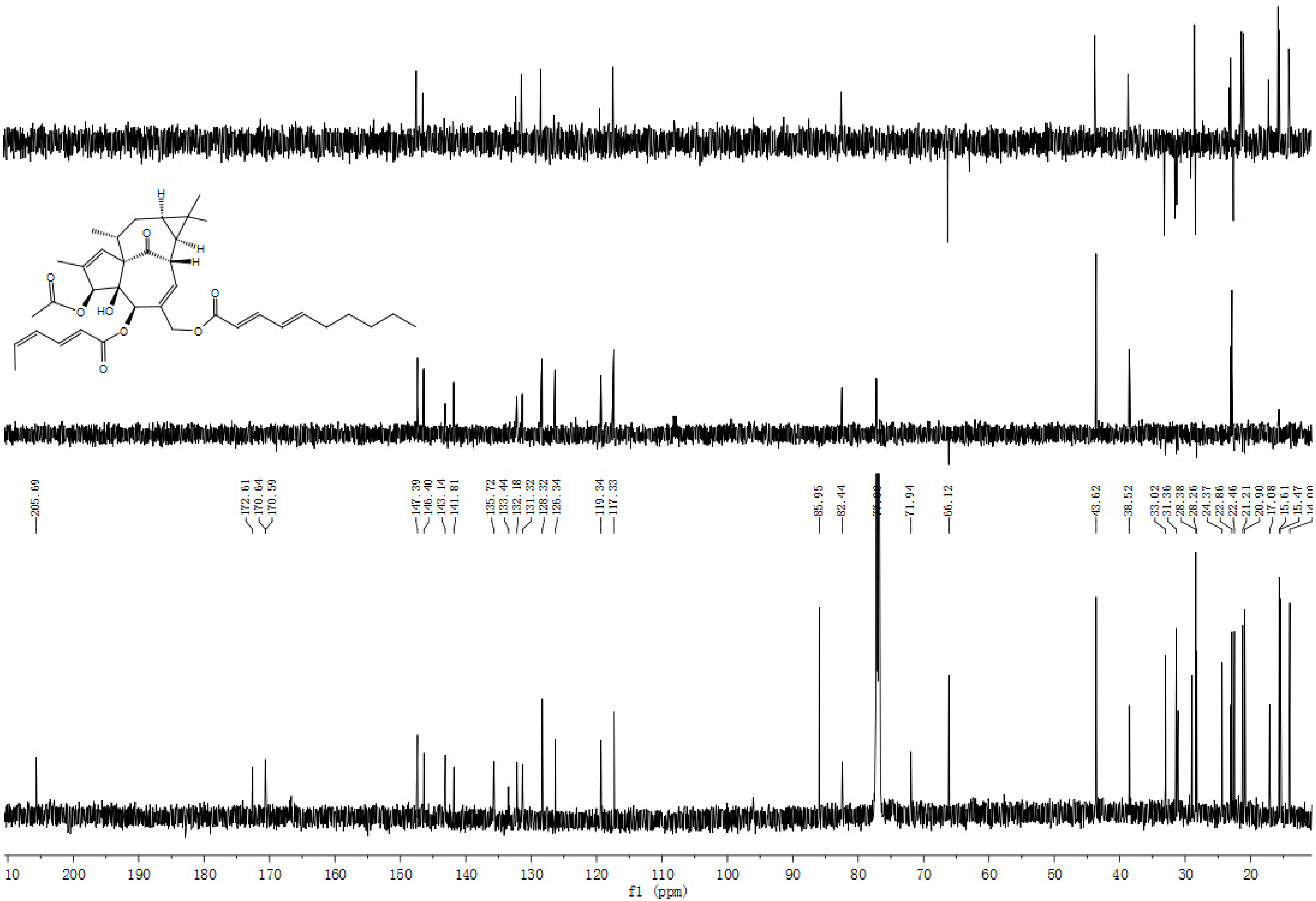


Figure S2. $^{13}$C NMR spectrum of sikkimenoid E (**1**)

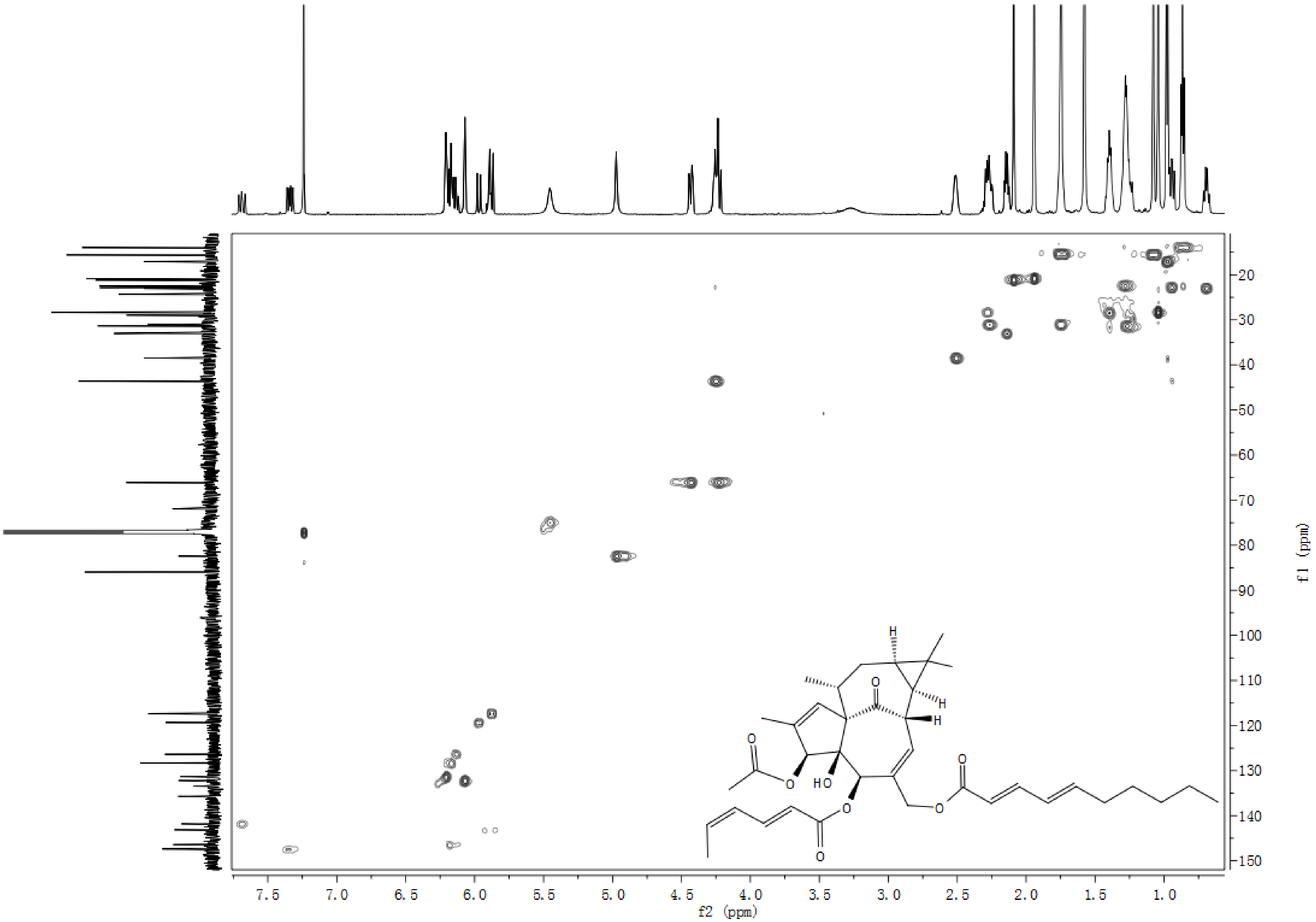


Figure S3. HSQC spectrum of sikkimenoid E (**1**)

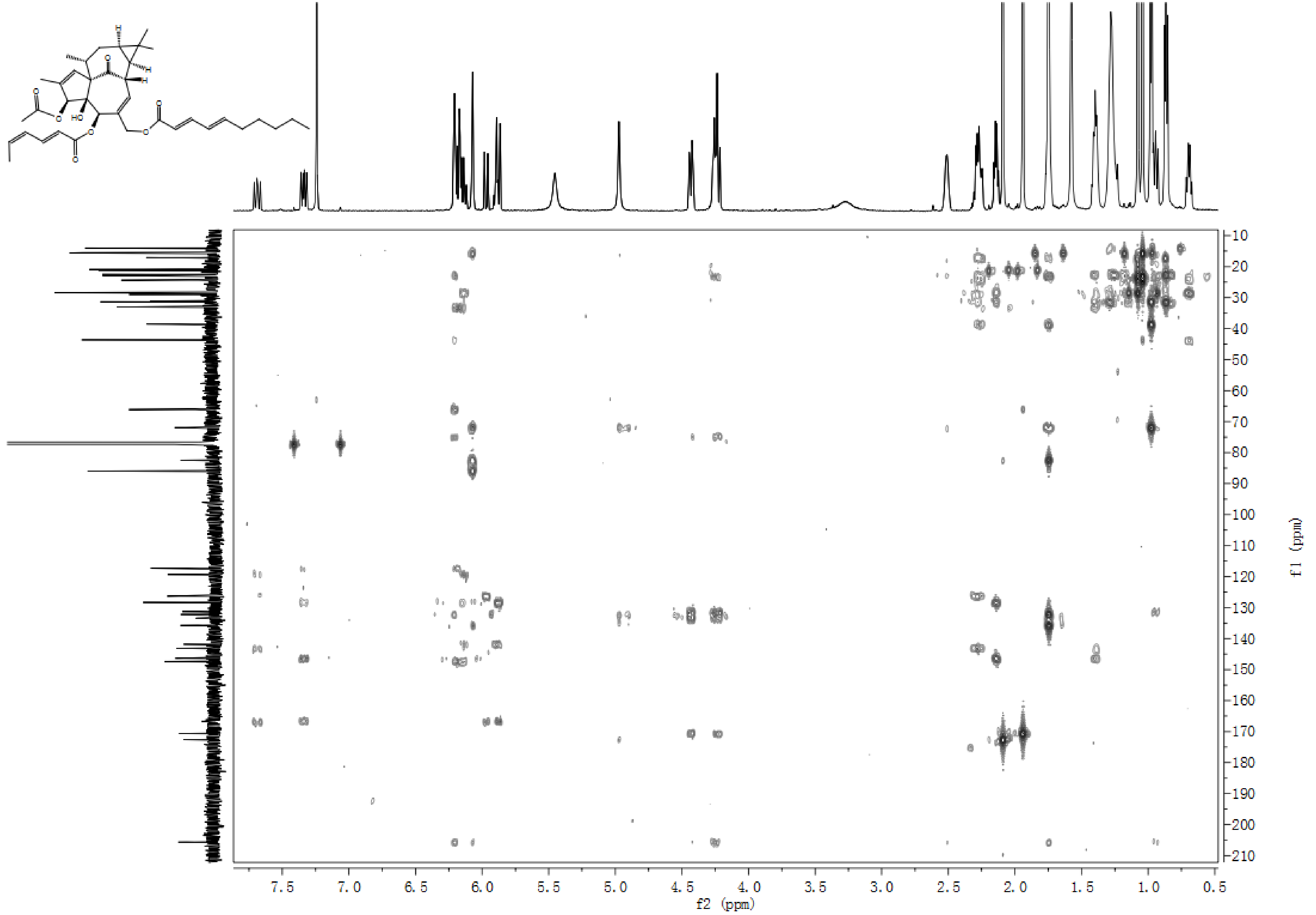


Figure S4. HMBC spectrum of sikkimenoid E (**1**)

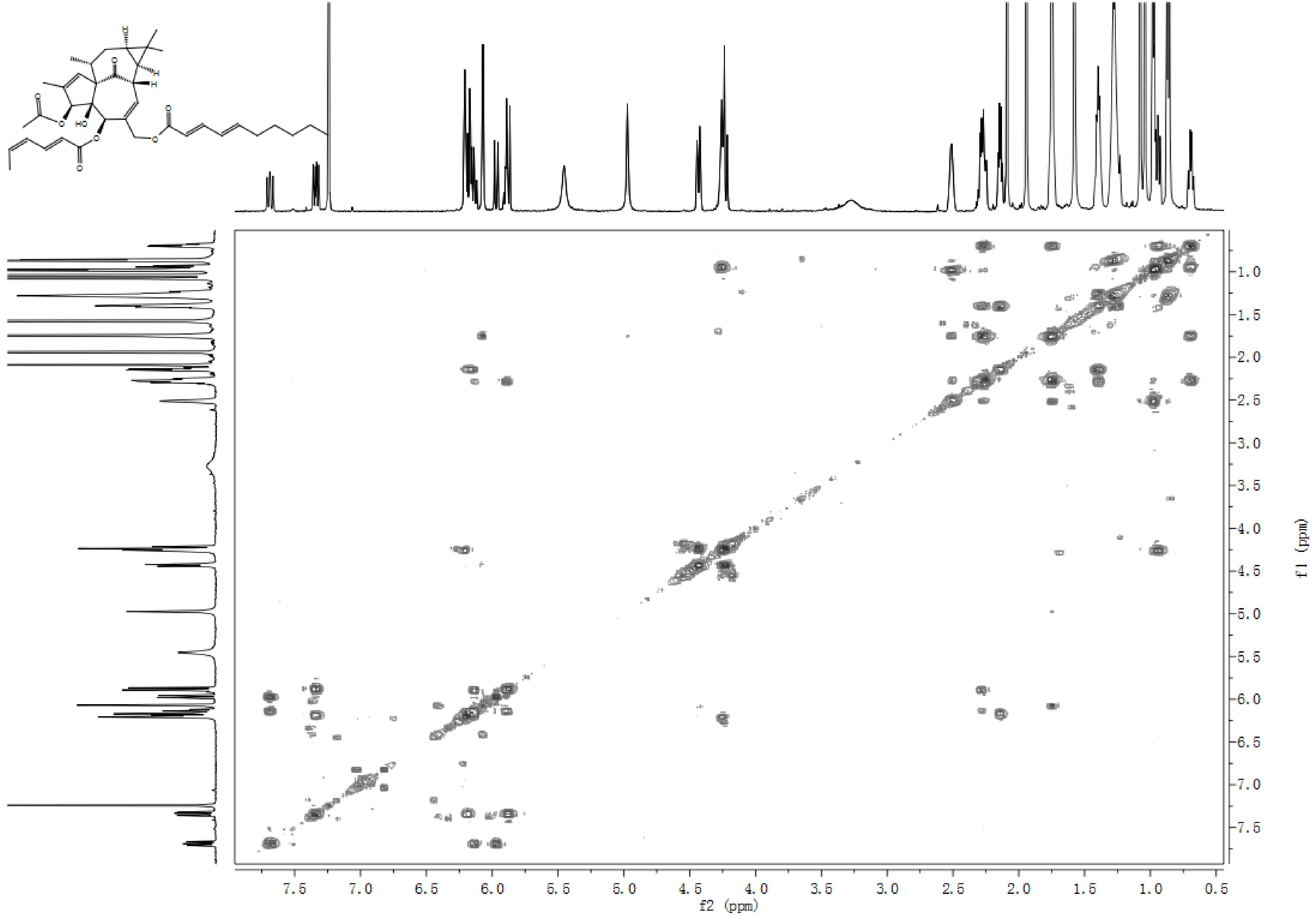


Figure S5. $^{1}H$–$^{1}H$ COSY spectrum of sikkimenoid E (**1**)

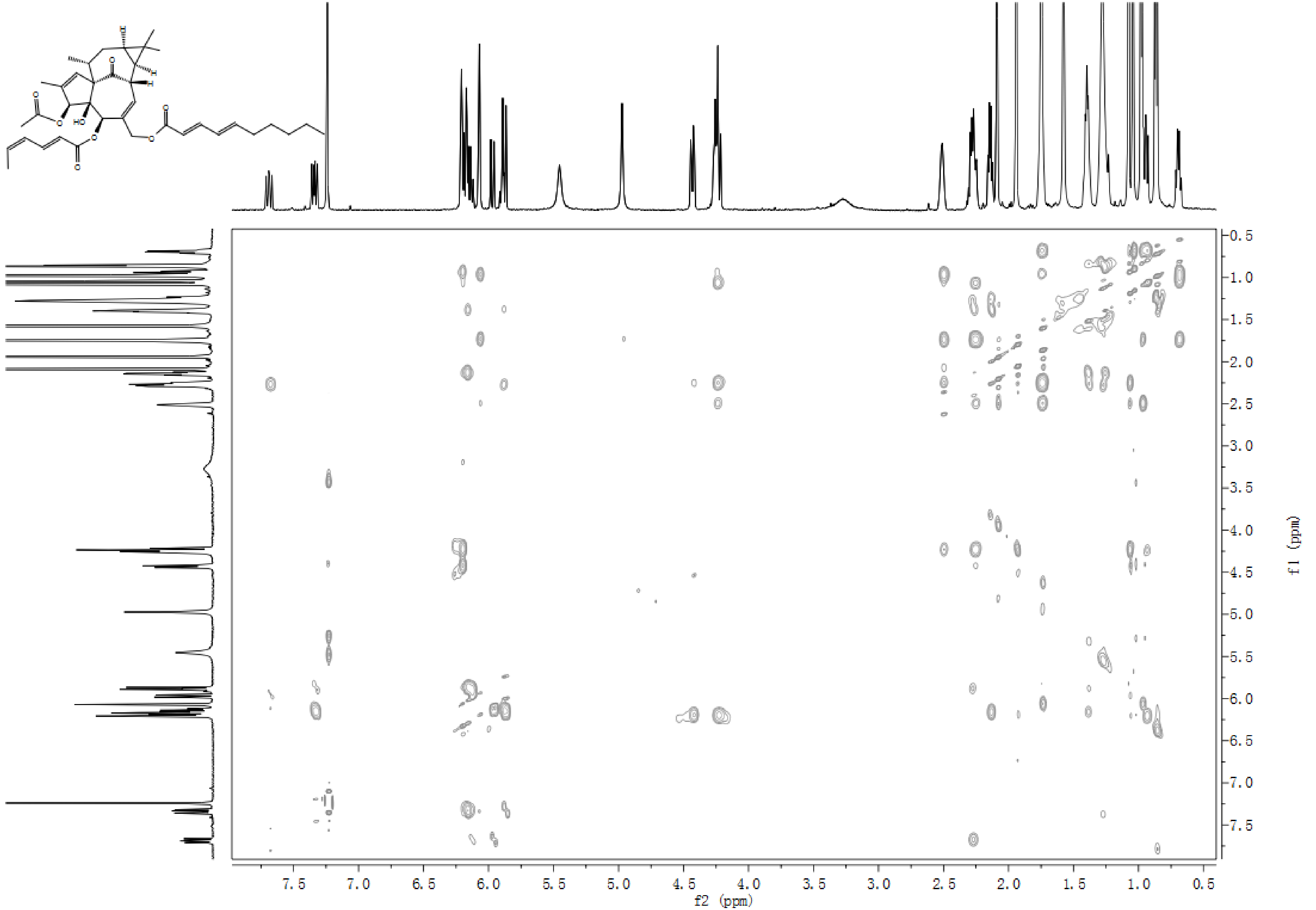


Figure S6. ROESY spectrum of sikkimenoid E (**1**)

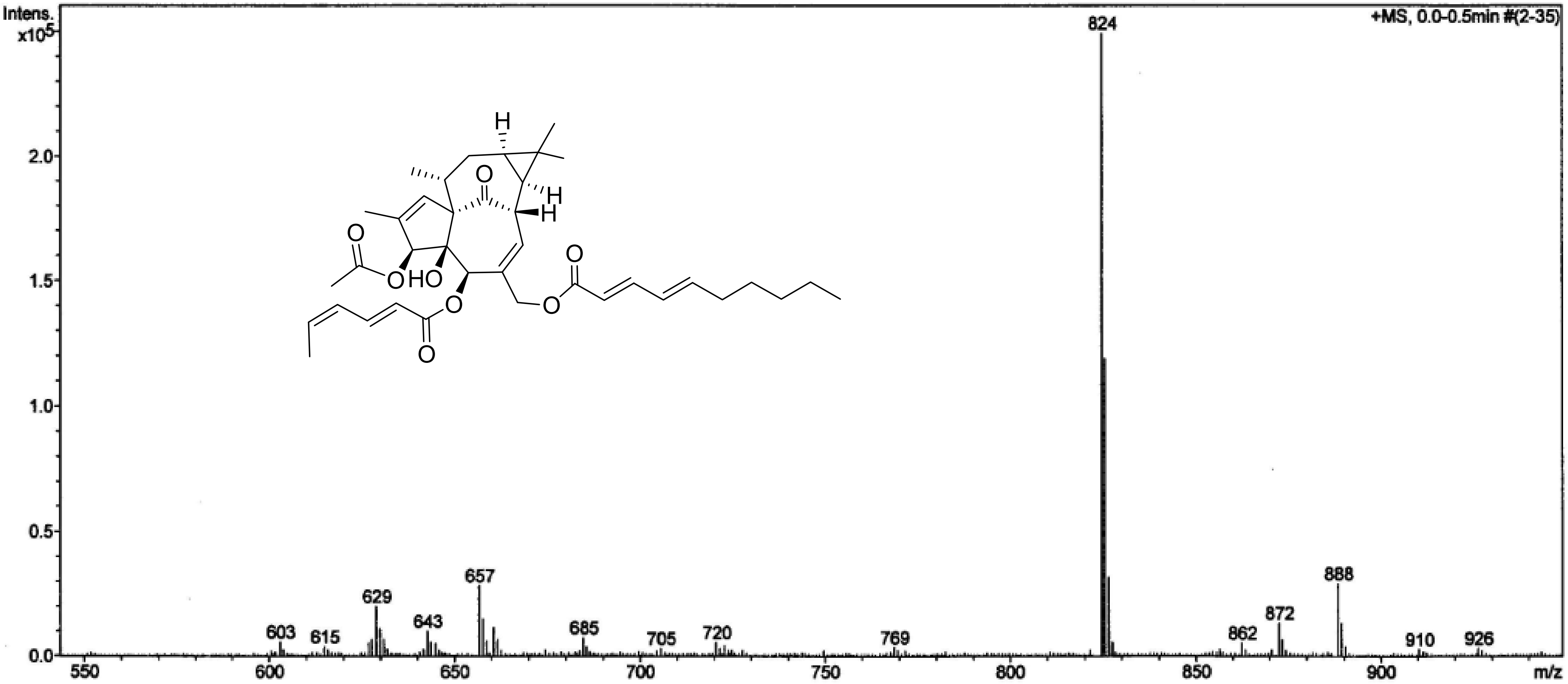


Figure S7. ESIMS spectrum of sikkimenoid E (**1**)

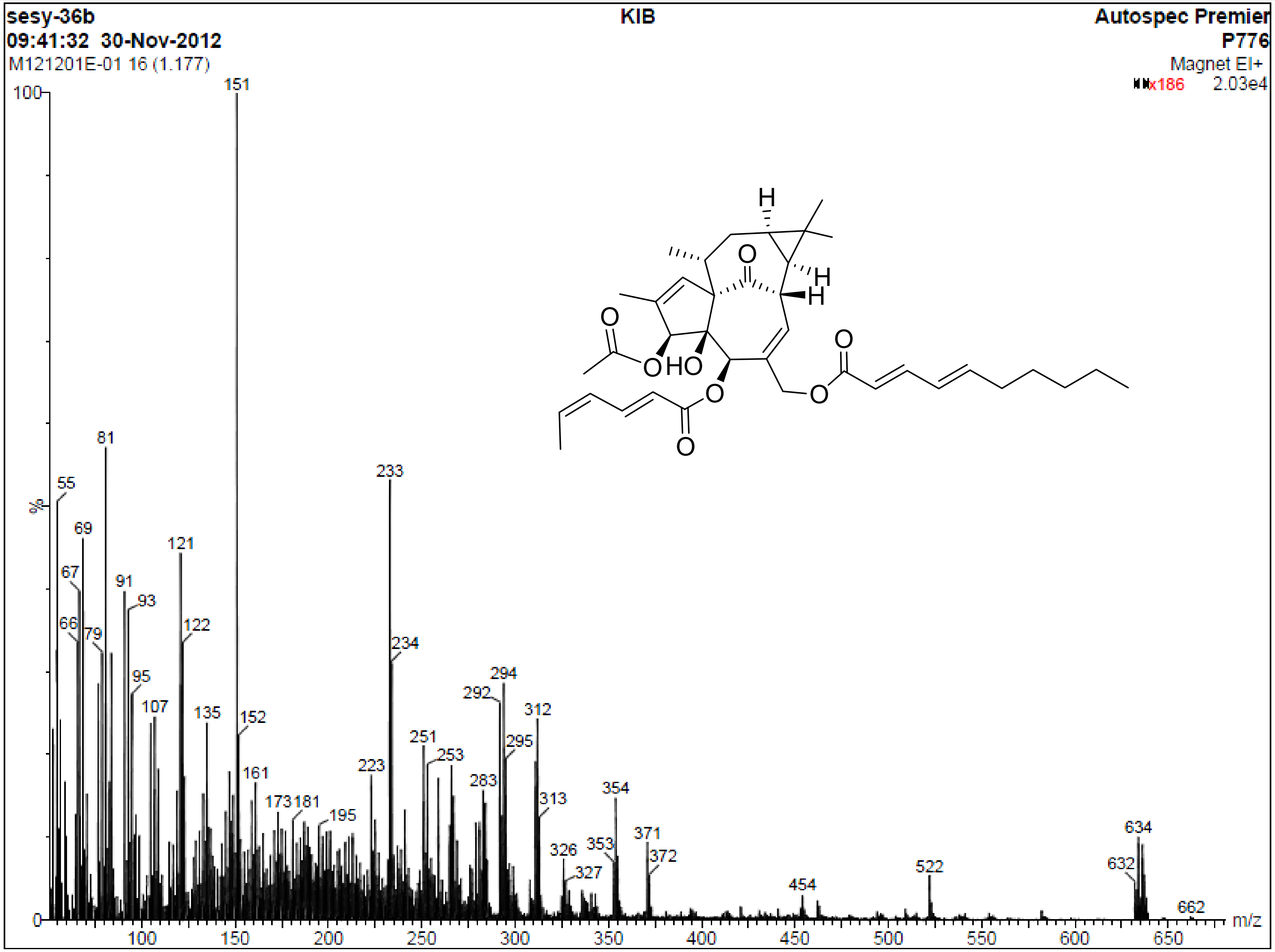


Figure S8. EIMS spectrum of sikkimenoid E (**1**)

# Single Mass Analysis

**Tolerance = 10.0 PPM / DBE: min = -10.0, max = 120.0**
**Selected filters: None**

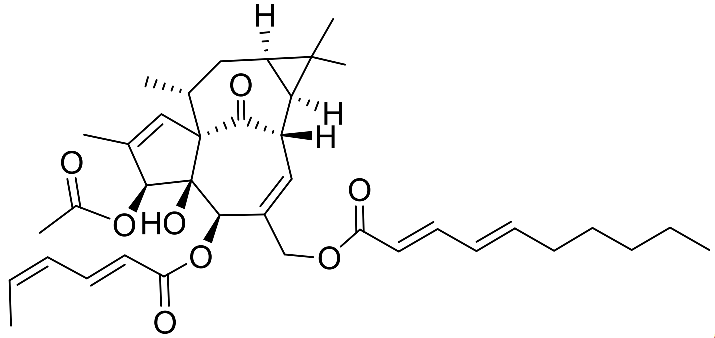


Monoisotopic Mass, Odd and Even Electron Ions
24 formula(e) evaluated with 1 results within limits (up to 51 closest results for each mass)
Elements Used:
C: 0-200 H: 0-400 O: 7-9

sesy-36b KIB Autospec Premier
09:35:20 30-Nov-2012 M121201EA-01AFAMM 21 (1.928) P776
Voltage EI+ 5

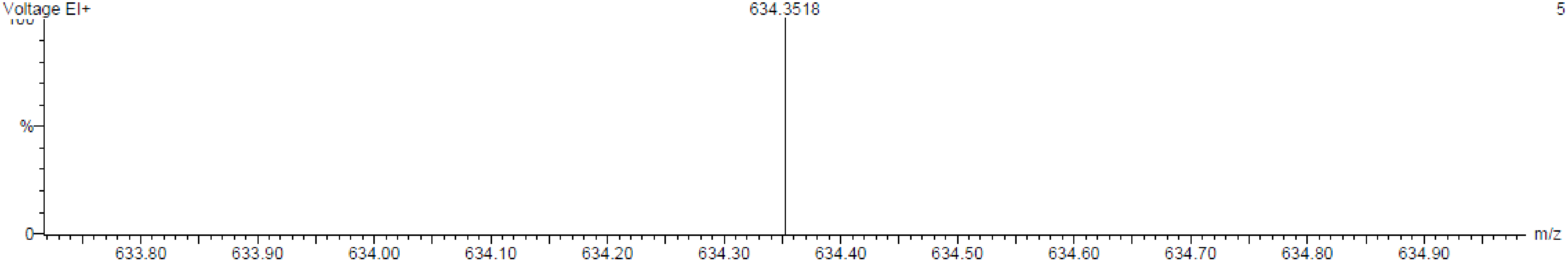


| | | | | | | |
|---|---|---|---|---|---|---|
| Minimum: | | | | -10.0 | | |
| Maximum: | | 100.0 | 10.0 | 120.0 | | |
| Mass | Calc. Mass | mDa | PPM | DBE | i-FIT | Formula |
| 634.3518 | 634.3506 | 1.2 | 1.9 | 14.0 | 5546025.5 | C38 H50 O8 |

Figure S9. HREIMS spectrum of sikkimenoid E

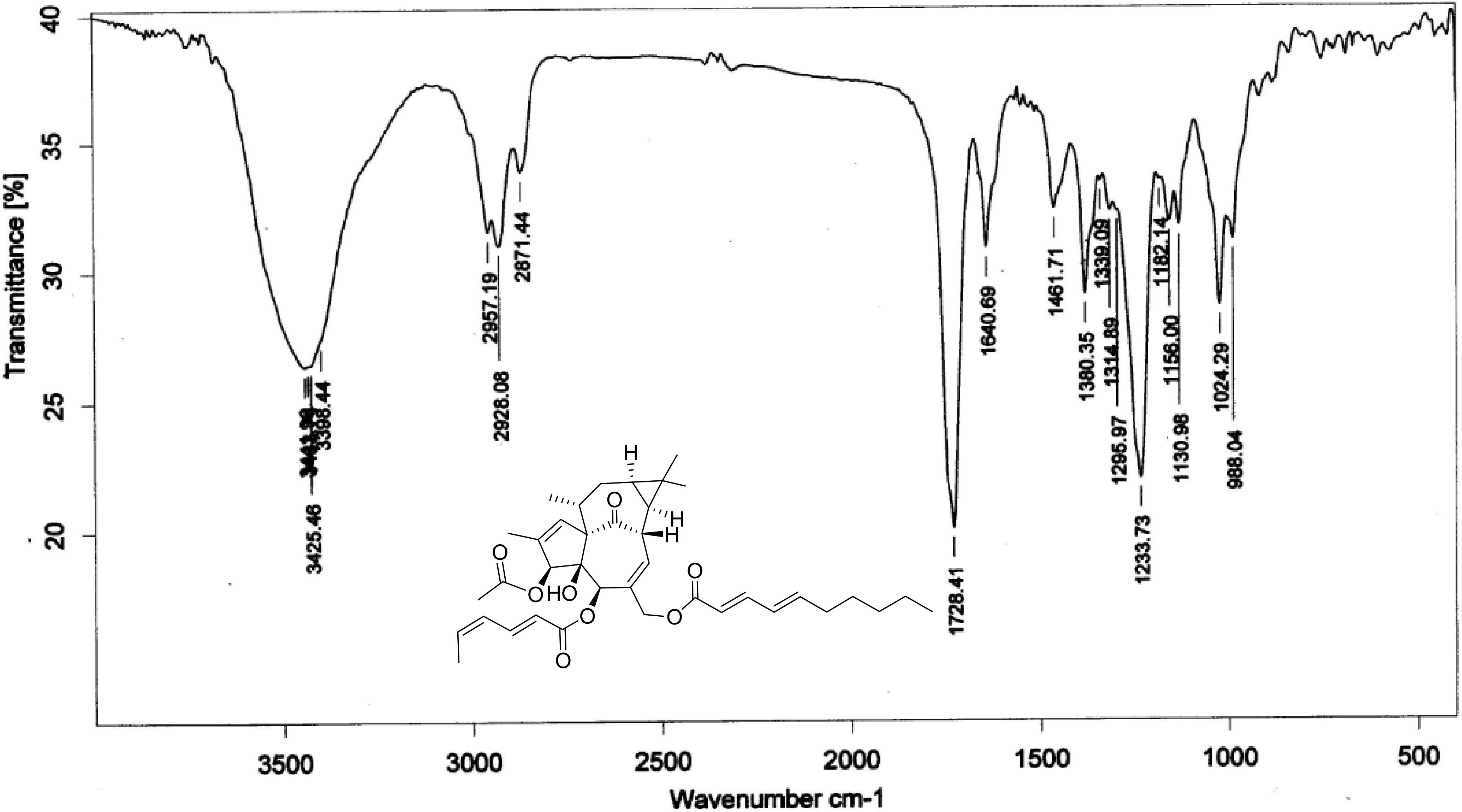


Figure S10. IR spectrum of sikkimenoid E (**1**)

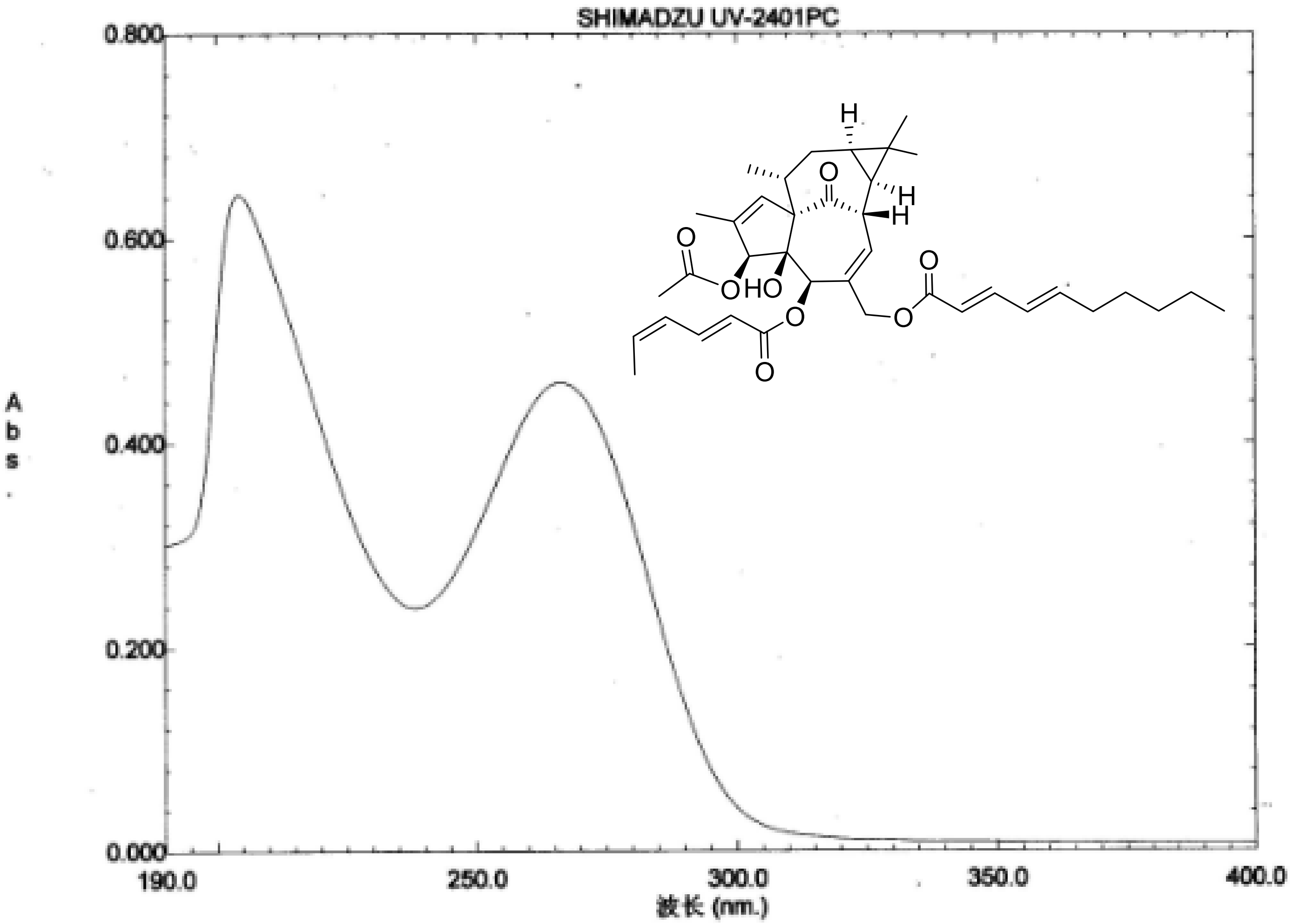


文件名: SESY-14A

SESY-14A

样品浓度：0.0190毫克/毫升
溶剂：甲醇

创建于: 17:45 12-10-15
数据: 原始

测量模式: Abs.
扫描速度: 中速
狭缝: 5.0
采样间隔: 0.2

| 否. | 波长 (nm.) | Abs. |
| --- | --- | --- |
| 1 | 266.40 | 0.4585 |
| 2 | 204.40 | 0.6427 |

Figure S11. UV spectrum of sikkimenoid E (**1**)

## Optical rotation measurement

Model : P-1020 (A060460638)

| No. | Sample | Mode | Data | Monitor<br>Blank | Temp.<br>Cell<br>Temp Point | Date<br>Comment<br>Sample Name | Light<br>Filter<br>Operator | Cycle Time<br>Integ Time |
|---|---|---|---|---|---|---|---|---|
| No.1 | 8 (1/3) | Sp.Rot | 39.8990 | 0.0079<br>0.0000 | 22.4<br>10.00<br>Cell | Mon Oct 15 15:09:36 2012<br>0.00198g/mlMeOH<br>SESY-14A | Na<br>589nm | 2 sec<br>10 sec |
| No.2 | 8 (2/3) | Sp.Rot | 37.3740 | 0.0074<br>0.0000 | 22.4<br>10.00<br>Cell | Mon Oct 15 15:09:49 2012<br>0.00198g/mlMeOH<br>SESY-14A | Na<br>589nm | 2 sec<br>10 sec |
| No.3 | 8 (3/3) | Sp.Rot | 35.3540 | 0.0070<br>0.0000 | 22.4<br>10.00<br>Cell | Mon Oct 15 15:10:03 2012<br>0.00198g/mlMeOH<br>SESY-14A | Na<br>589nm | 2 sec<br>10 sec |

+37.5421

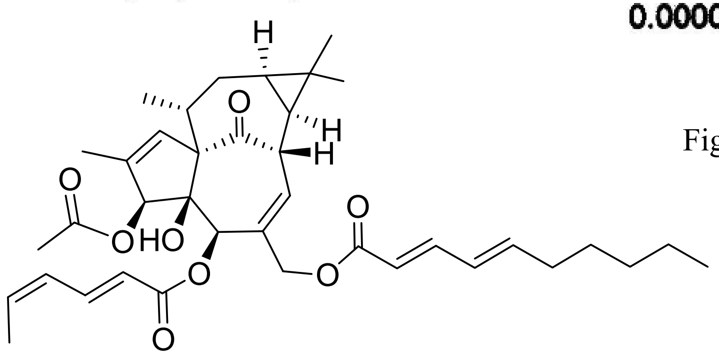


Figure S12. ORD spectrum of sikkimenoid E (**1**)

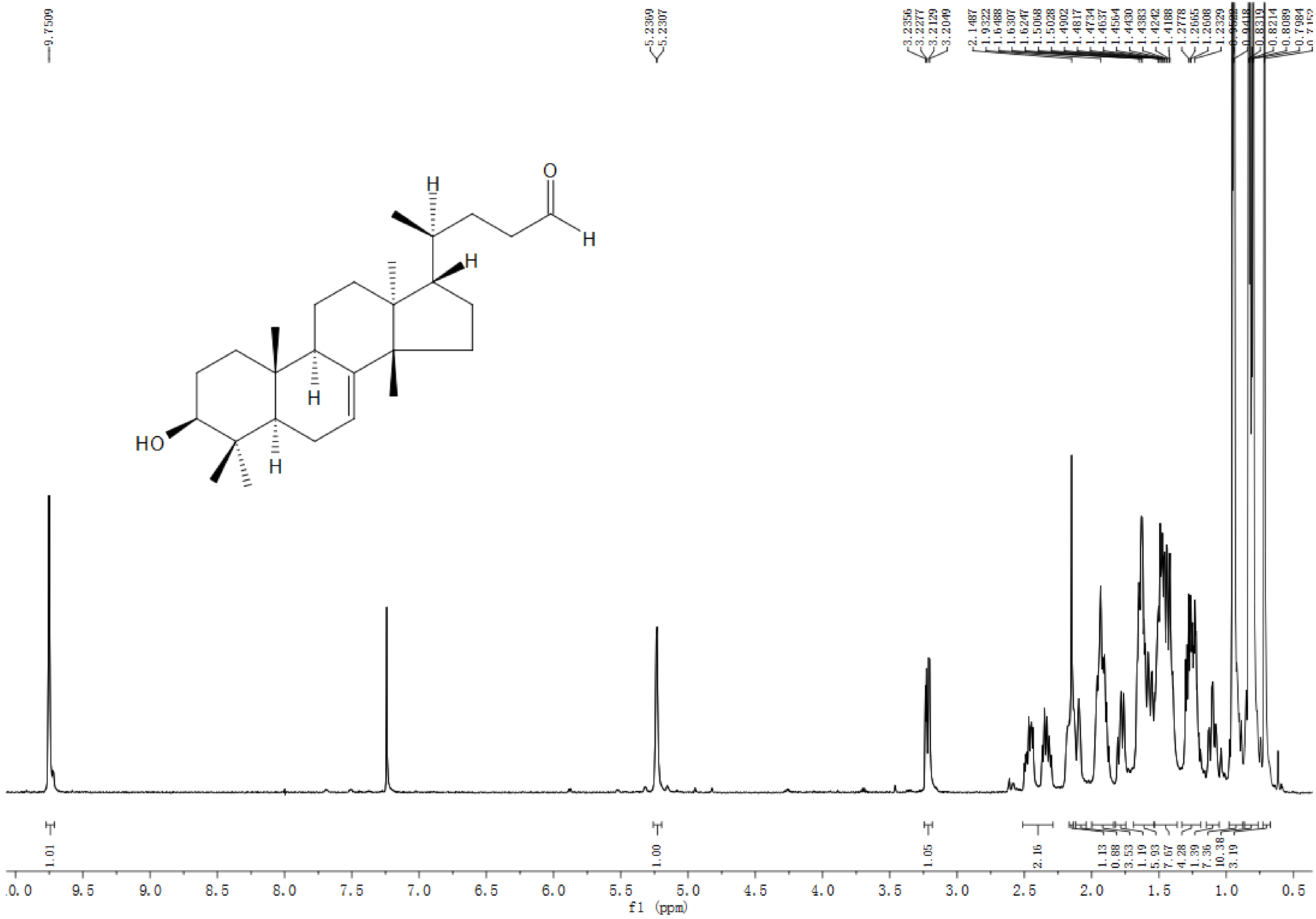


Figure S13. $^{1}$H NMR spectrum of sikkimenoid F (**2**)

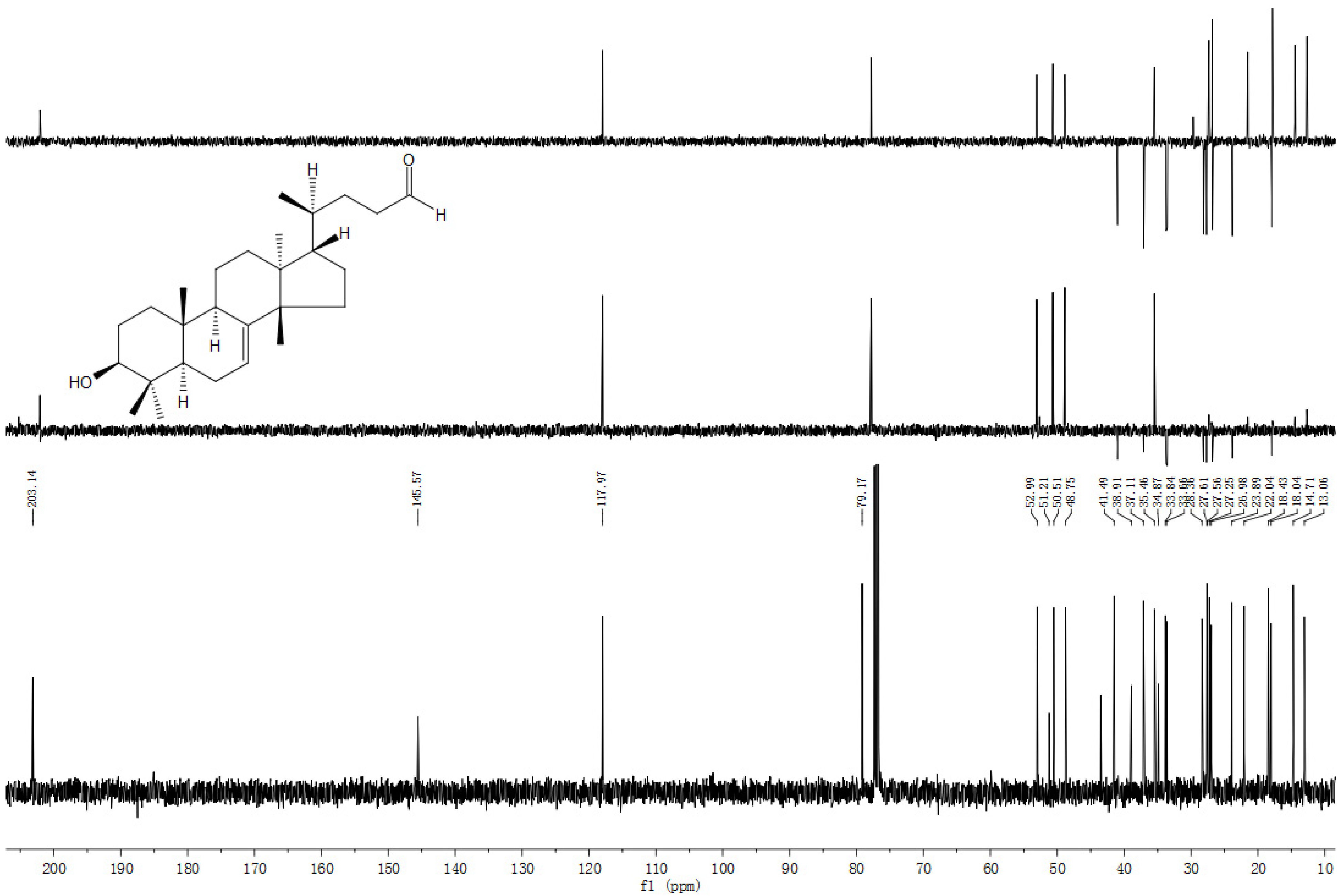


Figure S14. $^{13}$C NMR spectrum of sikkimenoid F (**2**)

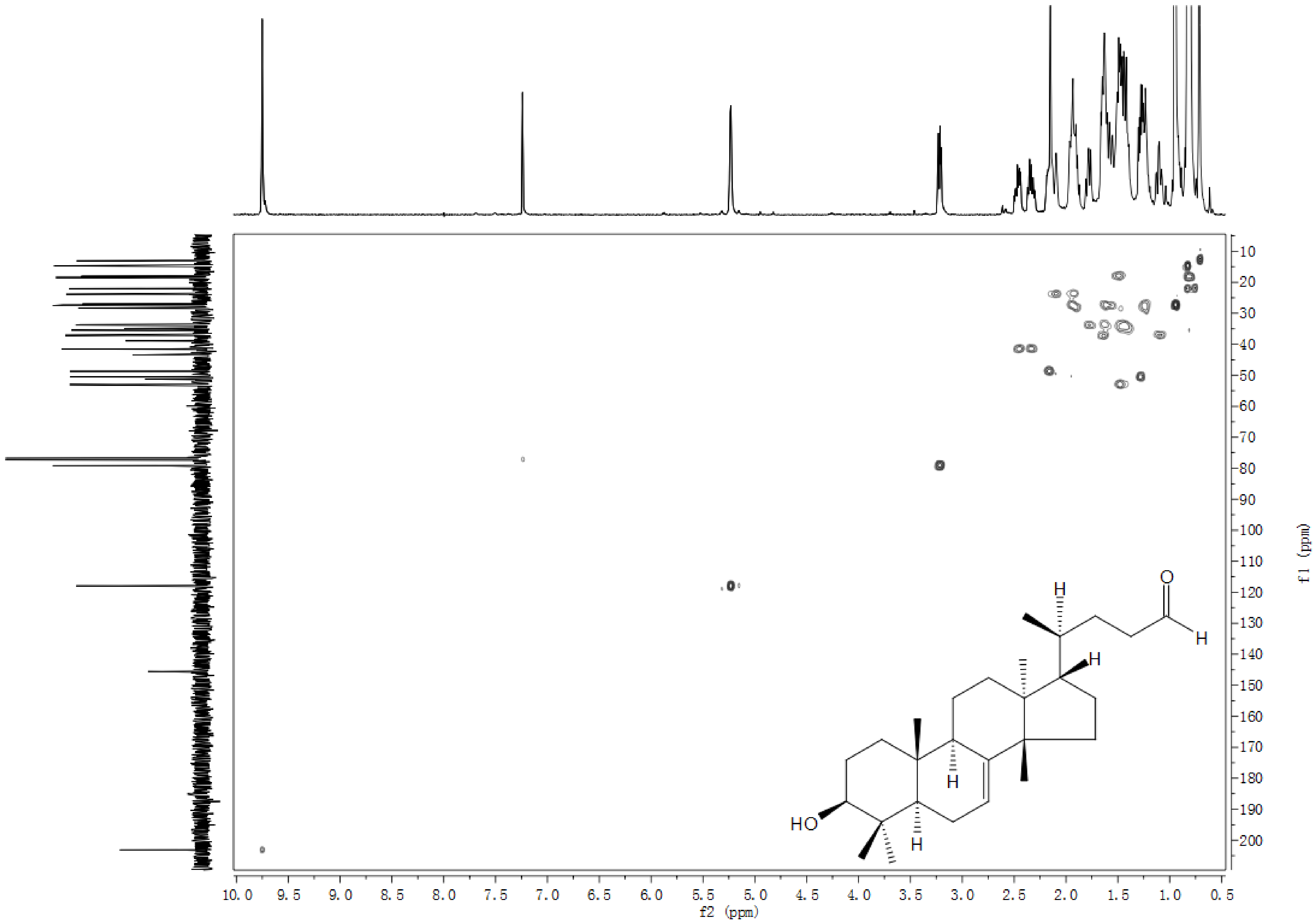


Figure S15. HSQC spectrum of sikkimenoid F (**2**)

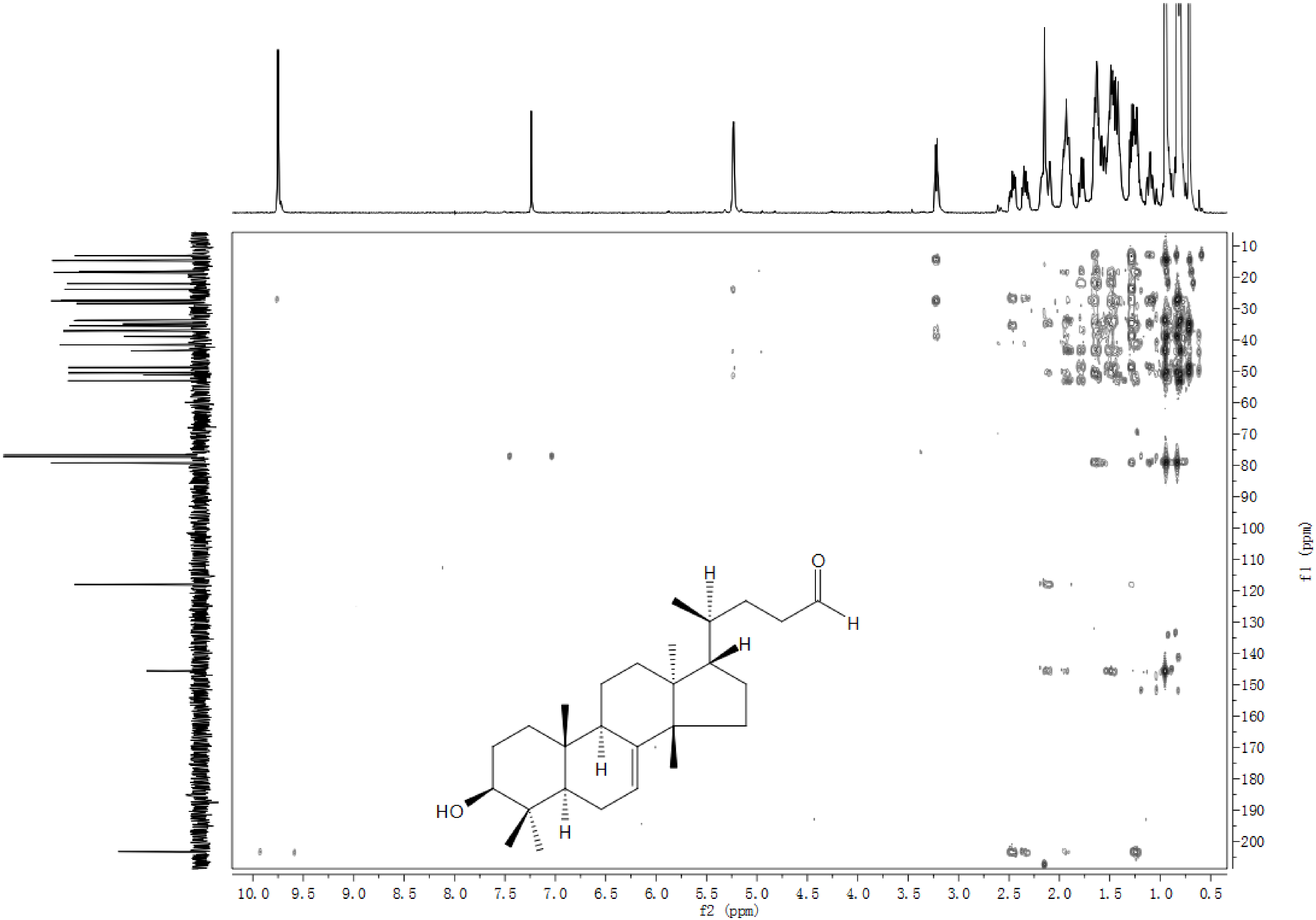


Figure S16. HMBC spectrum of sikkimenoid F (**2**)

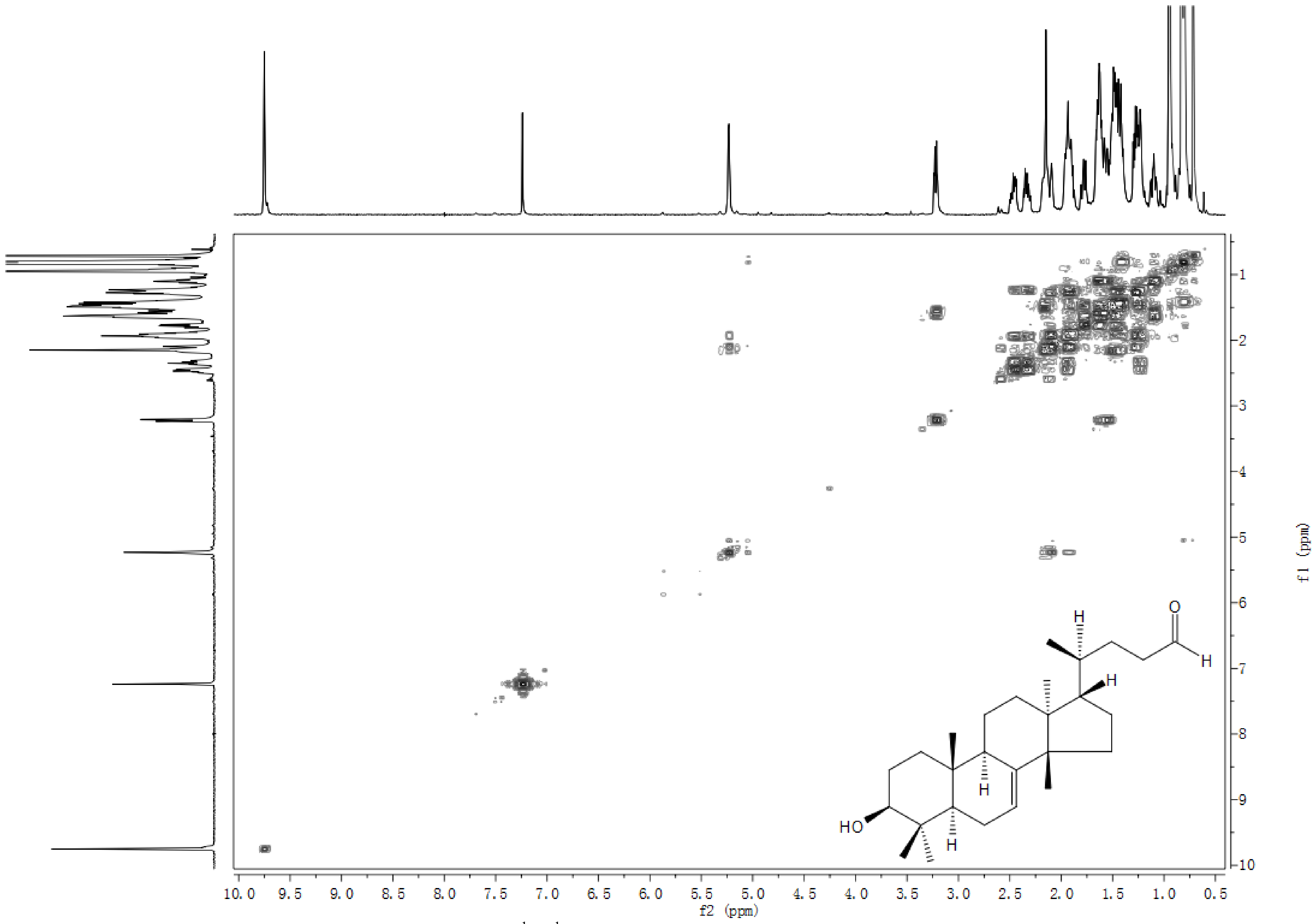


Figure S17. $^{1}H$–$^{1}H$ COSY spectrum of sikkimenoid F (**2**)

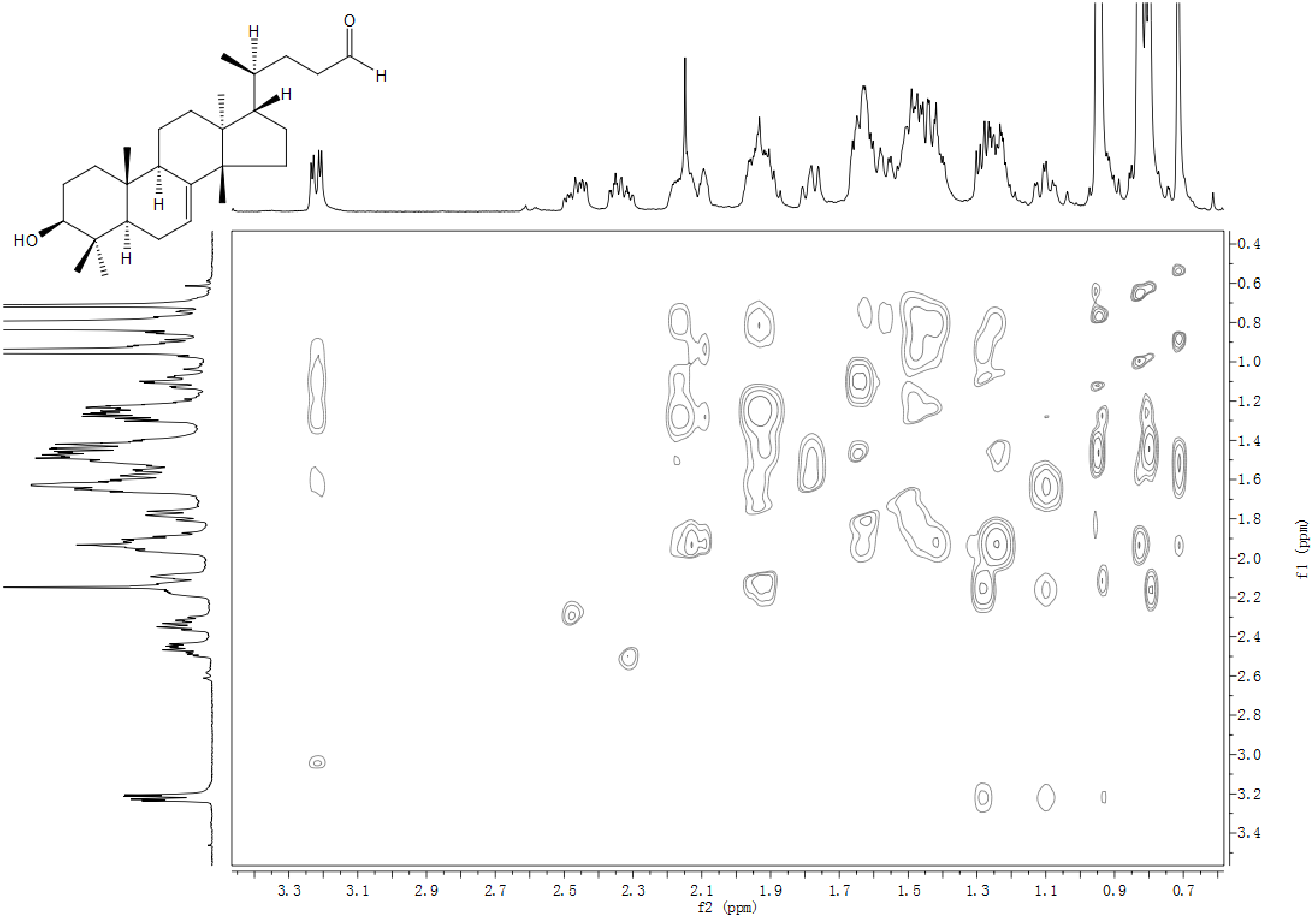


Figure S18. ROESY spectrum of sikkimenoid F (**2**)

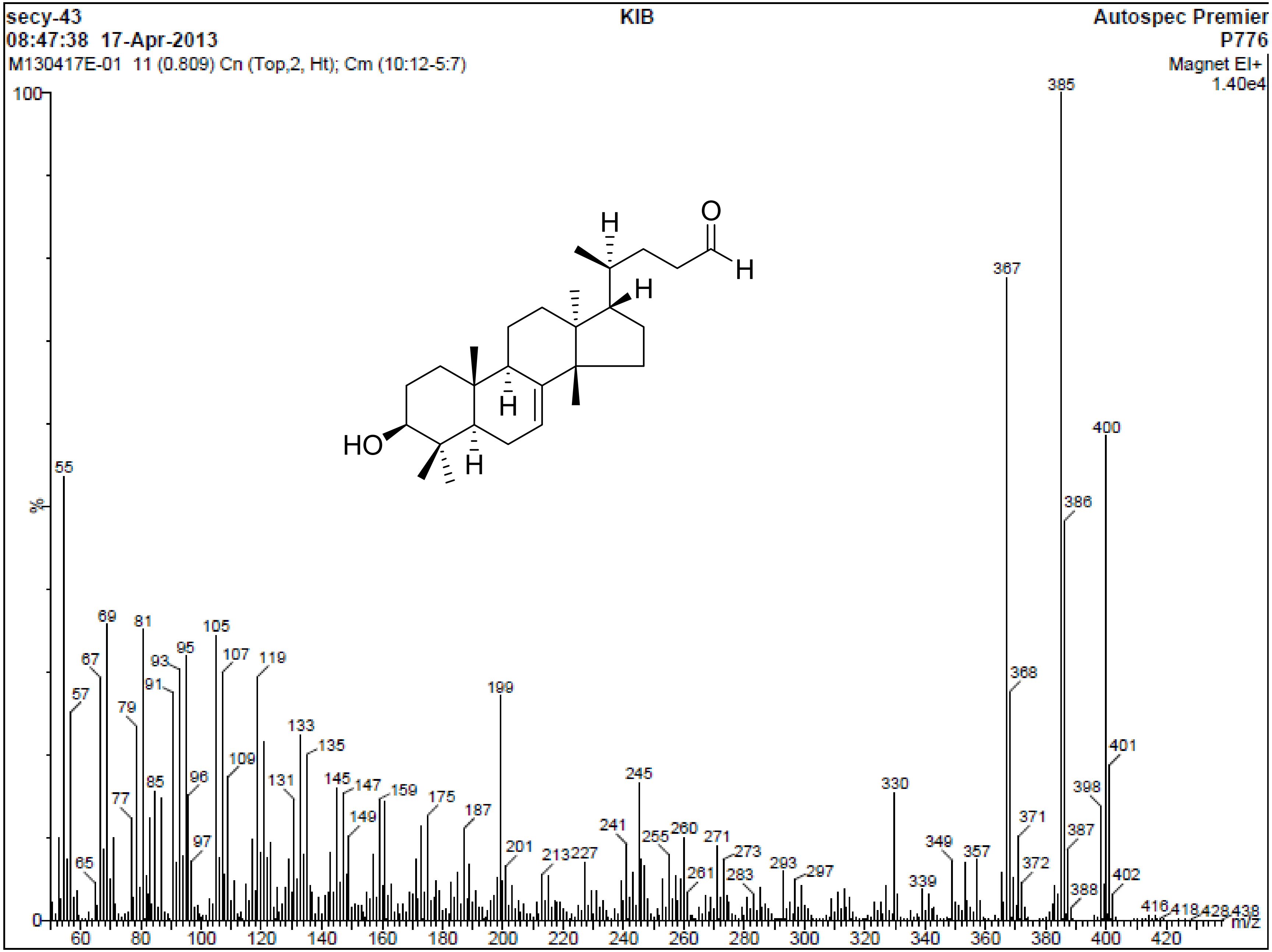


Figure S19. EIMS spectrum of sikkimenoid F (**2**)

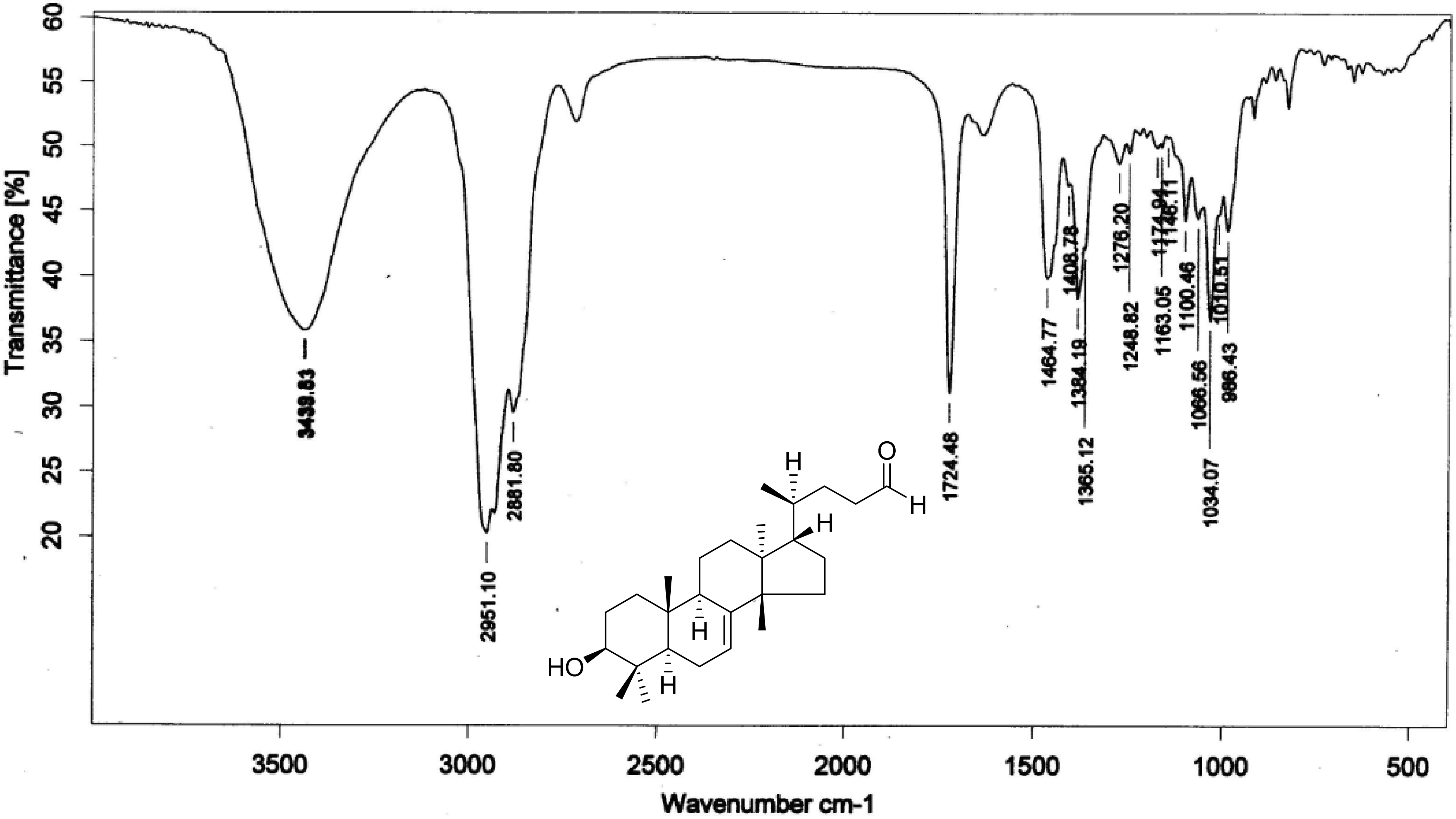


Figure S20. IR spectrum of sikkimenoid F (**2**)

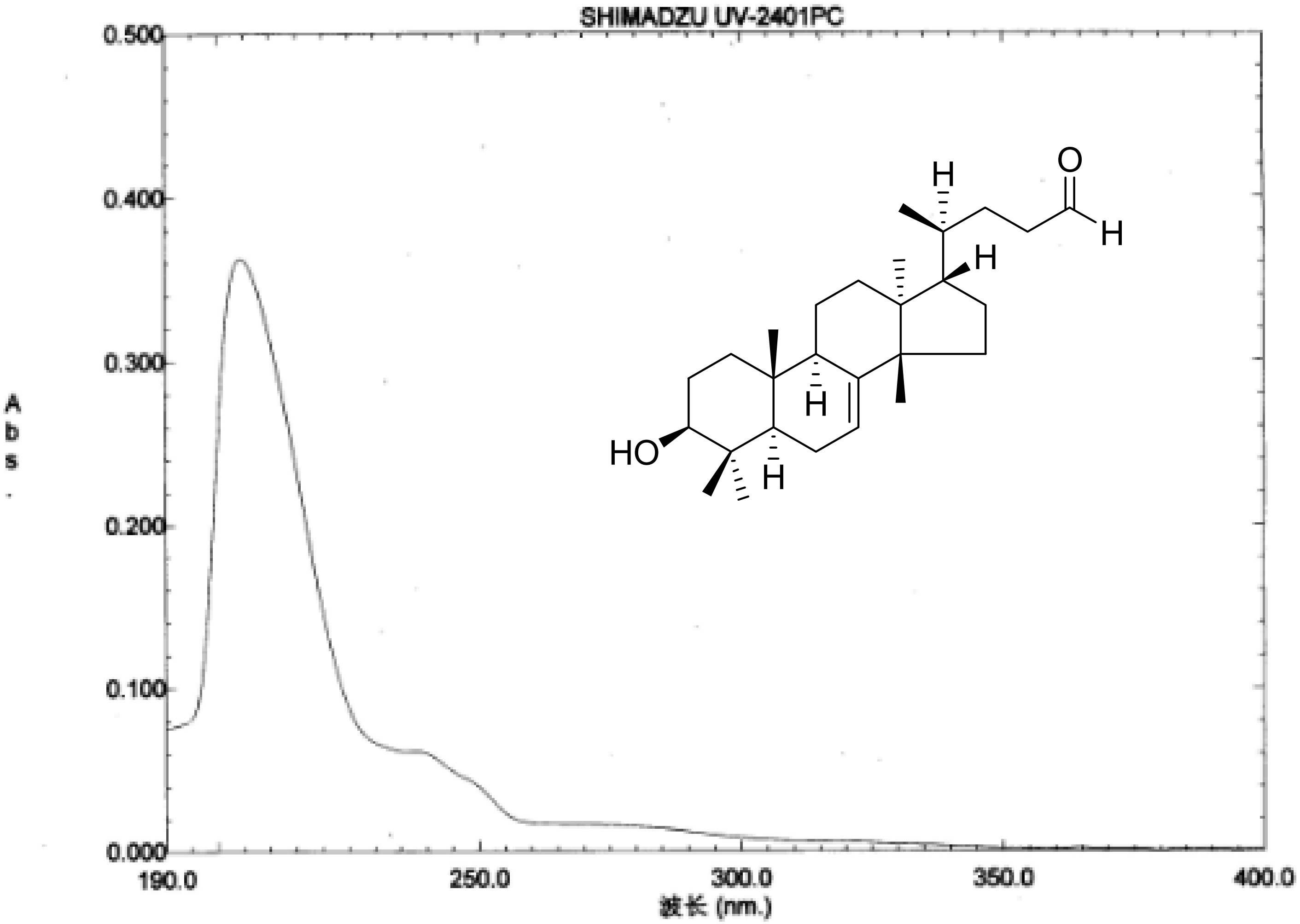


文件名:　SECY-43

SECY-43

样品浓度：0.0333毫克/毫升
溶剂：甲醇

创建于:　19:31　12-09-06
数据:　原始

测量模式:　Abs.
扫描速度:　中速
狭缝:　5.0
采样间隔:　0.2

| 否. | 波长 (nm.) | Abs. |
|---|---|---|
| 1 | 237.80 | 0.0612 |
| 2 | 204.60 | 0.3629 |

Figure S21. UV spectrum of sikkimenoid F (**2**)

Optical rotation measurement

Model : P-1020 (A060460638)

| No. | Sample | Mode | Data | Monitor<br>Blank | Temp.<br>Cell<br>Temp Point | Date<br>Comment<br>Sample Name | Light<br>Filter<br>Operator | Cycle Time<br>Integ Time |
|---|---|---|---|---|---|---|---|---|
| No.1 | 5 (1/3) | Sp.Rot | -16.2160 | -0.0270<br>0.0000 | 24.7<br>50.00<br>Cell | Thu Sep 06 16:59:18 2012<br>0.00333g/mlMeOH<br>SECY-43 | Na<br>589nm | 2 sec<br>10 sec |
| No.2 | 5 (2/3) | Sp.Rot | -15.1350 | -0.0252<br>0.0000 | 24.7<br>50.00<br>Cell | Thu Sep 06 16:59:31 2012<br>0.00333g/mlMeOH<br>SECY-43 | Na<br>589nm | 2 sec<br>10 sec |
| No.3 | 5 (3/3) | Sp.Rot | -17.3570 | -0.0289<br>0.0000 | 24.7<br>50.00<br>Cell | Thu Sep 06 16:59:45 2012<br>0.00333g/mlMeOH<br>SECY-43 | Na<br>589nm | 2 sec<br>10 sec |

-16.2362°

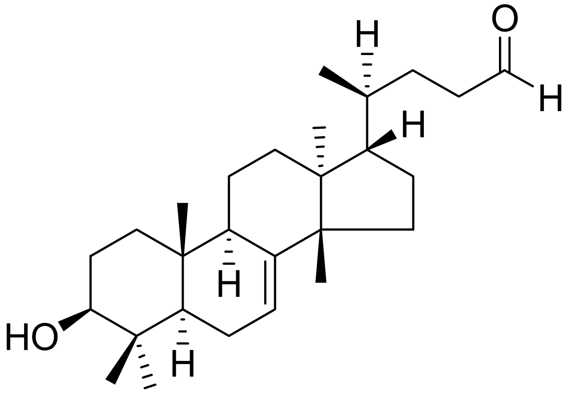


Figure S22. ORD spectrum of sikkimenoid F (**2**)
